# Molecular and functional properties of P2X receptors—recent progress and persisting challenges

**DOI:** 10.1007/s11302-012-9314-7

**Published:** 2012-05-01

**Authors:** Karina Kaczmarek-Hájek, Éva Lörinczi, Ralf Hausmann, Annette Nicke

**Affiliations:** 1 Molecular Pharmacology, RWTH Aachen University, Wendlingweg 2, 52074 Aachen, Germany; 2 Max Planck Institute for Experimental Medicine, Hermann Rein Str. 3, 37075 Göttingen, Germany

**Keywords:** Molecular, Functional properties, P2X receptors, Physiology, Pharmacology, Protein interactions, KO mice

## Abstract

ATP-gated P2X receptors are trimeric ion channels that assemble as homo- or heteromers from seven cloned subunits. Transcripts and/or proteins of P2X subunits have been found in most, if not all, mammalian tissues and are being discovered in an increasing number of non-vertebrates. Both the first crystal structure of a P2X receptor and the generation of knockout (KO) mice for five of the seven cloned subtypes greatly advanced our understanding of their molecular and physiological function and their validation as drug targets. This review summarizes the current understanding of the structure and function of P2X receptors and gives an update on recent developments in the search for P2X subtype-selective ligands. It also provides an overview about the current knowledge of the regulation and modulation of P2X receptors on the cellular level and finally on their physiological roles as inferred from studies on KO mice.

## Introduction

Adenosine 5’-triphosphate (ATP) is an essential macromolecule for all life forms and most likely evolved under the pre-biotic conditions prevalent on the primitive earth [1]. Research of the past 40 years has shown that ATP is not only a principal energy source and component of nucleic acids inside the cell but also plays a crucial role in intercellular communication [2]. This possibly oldest transmitter is involved in both fast and slow communication between cells by activating ionotropic P2X (ligand-gated ion channel receptors) or metabotropic P2Y (G-protein coupled receptors) receptor families [3–5]. P2X receptors are present in virtually all mammalian tissues and mediate a large variety of responses from fast transmission at central synapses, contraction of smooth muscle cells, platelet aggregation, and macrophage activation to proliferation and cell death, to only name a few [6]. After a short introduction of P2X receptor phylogeny, we will summarize the current information about their structure and function, synthesis and protein interactions, and focus on the recent developments in P2X receptor pharmacology. In addition, we will provide an overview of some physiological P2X receptor functions that are inferred from genetically modified mice and other in vivo models. For more detailed information on P2X receptor function in other systems, their distribution and signalling, a variety of excellent and comprehensive reviews are available [7–21].

## P2X receptors in different species

**Fig. 1:**
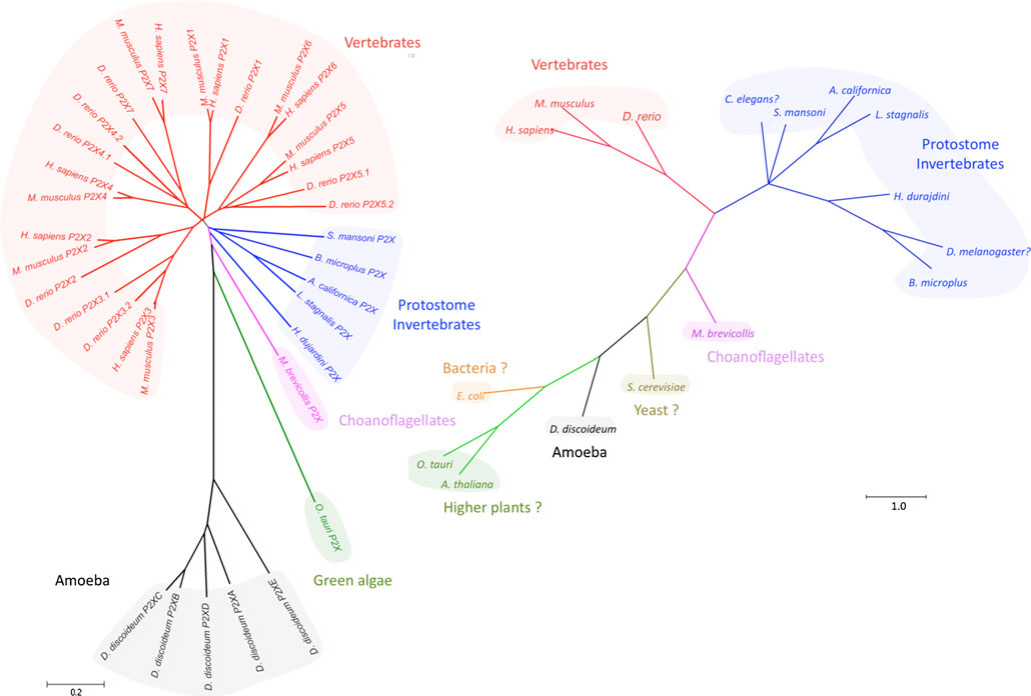
Evolutionary relationship of P2X receptors and common model organisms. *Left panel*: Unrooted neighbor-joining phylogeny of identified P2X protein sequences. The tree was constructed using the MEGA program (http://www.megasoftware.net/). The scale *bar* indicates the genetic distance in percent sequence divergence. *Right panel*: A phylogenetic tree showing the relationship between organisms in which P2X receptors are present and common model organisms in which P2X receptors have not been identified (indicated by question mark). The tree was created by hand and edited using the program Dendroscope (http://ab.inf.uni-tuebingen.de/software/dendroscope/) based on the information given in King et al. [35]. The following P2X receptor sequences were used: *D. discoideum* (XP_645378.1, XP_643830.2, XP_643831.1, XP_636768.1, XP_636957.2), *M. brevicollis* (EDQ92249.1), *S. mansoni* (CAH04147.1), *H. dujardini* (ACL14328.1), *B. microplus* (ADO64254.1), *A. californica* (AAR28669.1), *D. rerio* (NP_945333.1, NP_945334.1, NP_571698.1, NP_945337.2, AAH42317.1, AAI62598.1, NP_945336.1, NP_945335.1), *M. musculus* (AAF68968.1, AAK95327.2, AAH23089.1, AAC95601.1, AAK49936.1, NP_035158.2, NP_035157.2, AAI62774.1), *H. sapiens* (NP_002549.1, NP_733782.1, NP_002550.2, NP_002551.2, NP_002552.2, NP_005437.2, NP_002553.3), *O. tauri* (CAL54489.1). We thank Steve Ennion for providing the sequence of *L*. *stagnalis* and Henrik Krehenwinkel for phylogenetic analysis

Since 1994, seven mammalian P2X cDNAs (P2X1-P2X7) have been cloned [19, 22, 23]. Subsequently, P2X receptors were found to be also widely distributed among all vertebrate animals [24]. However, low sequence homology has made it difficult to determine potential homologues in invertebrate species. Since the identification of the first invertebrate P2X receptor in parasitic trematode *Schistostoma mansoni* [25], P2X receptor family members have also been discovered in more primitive life forms such as the unicellular amoeba *Dictyostelium discoideum* and the green algae *Ostreococcus tauri* [24, 26, 27], which is the smallest known free-living eukaryote [28, 29] (Fig. 1). Interestingly, *D. discoideum* P2X receptors are localized in the membrane of the intracellular contractile vacuole [27, 30]. These findings demonstrate that P2X receptors must not only be considered in the context of the plasma membrane but that at least phylogenetically older P2X receptors may have an intracellular ion channel function [27].

Evidence is accumulating that P2X receptors arose at the same time or even before the appearance of G-protein coupled P1 (adenosine) or P2Y receptors [31]. However, despite extensive bioinformatics efforts, no prokaryotic P2X receptor has been identified so far [24, 32], suggesting that structurally different ATP receptors evolved in bacteria and that the P2X receptors were not derived from a prokaryotic ancestor [31]. Considering the presence of the P2X channels in the photosynthetic *O. tauri* and the significance of ATP-mediated signalling in plant physiology [33, 34], it is also astonishing that there is no evidence for P2X counterparts in higher plants such as *Arabidopsis thaliana*. More sequenced genomes and experimental data are necessary to completely exclude the possibility of the existence of the P2X receptors homologues in prokaryotes and higher plants. Functional P2X receptors have been identified in unicellular choanoflagellates (*Monosiga brevicollis*), which are the closest known relatives of the animal kingdom [26, 35]. Despite this fact, P2X-like protein sequences appear to be absent in some commonly used model systems such as the yeast *Saccharomyces cerevisiae*, the nematode *Caenorhabditis elegans*, and the arthropods *Anopheles gambiae*, *Apis mellifera*, and *Drosophila melanogaster* [10, 24, 25, 36]. The absence of genes coding for P2X receptors in these animal groups is in contrast to the potent pharmacological actions of nucleotides in these species, suggesting that novel receptors are still to be discovered [31]. Indeed, a first arthropod P2X receptor (BmP2X from the cattle tick *Boophilus microplus*) has been described most recently [37]. The presence of P2X receptors in other members of this group could be anticipated, since functional P2X receptors have been identified in the tardigrade species *Hypsibius dujardini* [36] which, together with arthropods and nematodes, belongs to the common superphylum Ecdysozoa. These new findings support the postulate that the P2X genes have not been lost in an ancestor of the Ecdysozoa but rather disappeared independently in nematodes and maybe some arthropods before they diverged from Tardigrada [36].

Nevertheless, it is still not clear if other, not sequenced members of nematodes and arthropods are also void of P2X channels. Notably, P2X homologues have also been identified in the CNS of *Aplysia californica* and *Lymnaea stagnalis*, which are members of the superphylum Lophotrochozoa, a sister clade to the Ecdysozoa [38, 39]. According to the present state of knowledge, it seems that the development of the seven mammalian P2X genes was a relatively recent phenomenon and occurred after the branching between vertebrates and invertebrates (Fig. 1) [36]. Taken together, further identification of P2X receptors in various organisms, especially primitive ones, will be necessary to better understand the evolutionary gaps mentioned above and eventually trace the phylogenetic history of P2X receptors. Moreover, new sequences from different P2X family members provide useful information to decipher their structure–function relationships.

## Molecular structure and function of P2X receptors

### Primary structure and subunit topology

The seven cloned human and rat P2X subunits are between 379 (rat P2X6) and 595 (rat and human P2X7) amino acids long and share 35–54% sequence identity. All have a common topology with two transmembrane (TM) domains, a large extracellular ligand binding loop, and intracellular N and C termini. The extracellular domain connecting the two TMs constitutes the largest part of the polypeptide. An important feature is the presence of ten Cys residues conserved among all vertebrate receptors and bound in five disulfide bridges [40, 68]. In addition, all rat subunits contain three to six consensus sequences for N-linked glycosylation (see section “Synthesis and trafficking of P2X receptors”). The N termini are similar in length (20–30 amino acids) and contain a consensus site for protein kinase C (PKC) phosphorylation [41]. The C termini differ in length between 26 (P2X6) and 239 (P2X7) amino acids and exhibit only sequence relatedness for the first 25 amino acid residues, indicating that they might serve subunit specific properties [21]. They contain several motifs involved in trafficking and stabilization of the receptors in the plasma membrane and specific protein interactions (for further details, see sections “Synthesis and trafficking of P2X receptors”and “Regulation and protein interactions of P2X receptors”). The primary sequence of P2X receptors shares no significant homology with other ligand-gated ion channels, ATP-binding proteins, or other known proteins.

### Quaternary structure and subunit assembly

Early electrophysiological measurements in dorsal root ganglion (DRG) neurons predicted that there are at least three ATP molecules needed to open a P2X channel [42]. Subsequent single-channel analysis of P2X2 receptors supported this idea [43]. The first biochemical evidence for a trimeric organization of P2X receptor channels came from cross-linking studies and blue-native PAGE analysis of P2X1 and P2X3 receptors heterologously expressed in oocytes of *Xenopus laevis* [44]. The trimeric architecture was confirmed by atomic force microscopy [45], electron microscopy and single particle analysis [46, 47], and finally, by crystallization of the first P2X receptor, the P2X4 subtype from zebrafish (zP2X4) [48]. Although P2X receptors share the TM topology and trimeric quaternary structure with the epithelial Na^+^ channels (ENaC)/DEG (epithelial sodium channels/degenerin) superfamily of proteins they show no significant amino acid sequence relationships or similarities in the extracellular domain [48–50].

Heterologously expressed P2X receptors have been characterized extensively in terms of their biophysical and pharmacological properties (for a recent review, see [12]), and there is good evidence for homomeric P2X1, P2X2, P2X3, P2X4, and P2X7 receptors in native tissues [12]. However, P2X receptor properties also often do not match with those observed in native tissues, suggesting that P2X receptors occur naturally as both homo- and hetero-oligomers [51]. Indeed, only the P2X7 subunit appears unable to form heterotrimeric channels with other subunits [52, 53]. In contrast, the P2X6 receptor is the only subunit virtually unable to form homo-oligomers [45, 54]. In addition to heteromerization, splice variants and the presence of more than one functional P2X subtype in many cell types can contribute to the diversity of P2X receptor signalling. For P2X5 receptors, which occur in humans as a non-functional splice variant, species-specific differences in heterologous expression efficiency and functional properties such as ion permeability are observed [55–62]. Regarding heteromeric receptors, the best evidence and most comprehensive data exist for P2X2/3 receptors. For these, a subunit stoichiometry of one P2X2 and two P2X3 subunits has been demonstrated [63, 64], and their presence and importance in native tissues has been shown in numerous studies, e. g., [8, 10, 65, 66] (see also section “P2X3”). Nevertheless, there is also good evidence that, in acutely isolated cortical astrocytes, heteromeric P2X1/5 receptors mediate the ATP-elicited currents, as these match the pharmacologic and kinetic properties of heterologously expressed P2X1/5 receptors [67]. Hardly any of the four other heteromeric subunit combinations characterized in heterologous expression systems (P2X1/2, P2X1/4, P2X2/6, P2X4/6) has been convincingly verified in native tissues. For details on their functional and pharmacological properties, see Coddou et al. and Nicke et al. [12, 51].

### Crystal structure of the P2X receptor

**Fig. 2:**
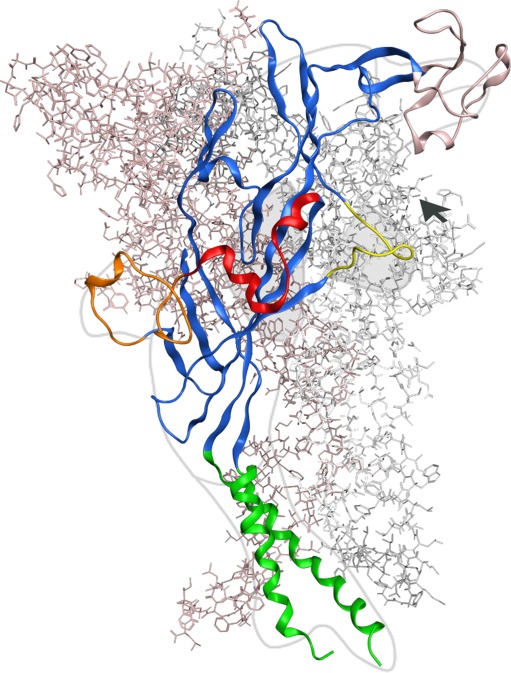
Homology model of the homomeric P2X2 receptor. The homotrimeric rP2X2 receptor structure is shown from the side, i.e., parallel to the membrane plane. Two subunits are shown as *pink or gray* sticks; one subunit is *highlighted as ribbon representation* with depiction of α-helices, β-sheets, and coil regions. The *dolphin-like shape* of this single subunit [48] (body, *blue*; fluke, *green*; head, *pink*; dorsal fin, *orange*; right flipper, *red*; left flipper, *yellow*) is emphasized by an *overlay* of a *grey dolphin* *cartoon*. The *arrowhead* indicates one of the three possible ATP binding pockets. The rP2X2 receptor homology model based on the X-ray structure of the zP2X4.1 receptor was generated using the MOE2008.10 software. For further details, see [93]. The figure was generated and kindly provided by Achim Kless, Grünenthal GmbH, Global Drug Discovery

A major breakthrough in P2X receptor research was the crystallization of the first P2X receptor by Kawate et al. [48], which provided a fundamentally new ion channel structure. Screening a variety of P2X receptor isoforms, a zP2X4.1 receptor containing three point mutations (C51F/N78C/N187R) and lacking the N and C termini (ΔP2X4-B, PDB entry 3H9V) was found optimal for crystallization and could be resolved at 3.1 Å. The homotrimeric receptor resembles a chalice, with the large extracellular domain protruding ~70 Å above the membrane plane and displays a right-handed twist if seen from the cytoplasmic side. The TM region has an hourglass shape formed by the six helices. Due to weak electron densities in the inward facing thirds of TM1 and TM2, these were less well resolved, resulting in unclear side chain orientations in these regions. The zP2X4 structure has been compared with the shape of a dolphin, in which the TM helices and the extracellular region form the flukes and the upper body, respectively (Fig. 2). Attached to the body domain, a head domain, a dorsal fin, and right and left flippers have been defined. The body domain is structurally rigid, characterized by a β-sandwich motif, with extensive contacts between the sandwich-forming β-sheets. The structure confirmed the existence of the five proposed disulfide bridges [40, 68], three of which are located in the head domain.

### Ligand binding sites

#### ATP binding

Unlike other ATP-binding proteins, P2X receptors lack consensus sequences for ATP coordination [20]. Before the P2X4 crystal structure became available, extensive studies on P2X1, P2X2, P2X3, and P2X4 receptors [15, 16, 69–74] employed mutagenesis-based approaches to localize the agonist binding site (for recent reviews, see Coddou et al. and Evans [12, 16]). These studies led to the conclusion that the ATP binding pocket is generally conserved within the P2X receptor family, and positively charged amino acid residues coordinate the negatively charged phosphate oxygens of ATP. In particular, Lys68, Lys70, Arg292, and Lys 309 (P2X1 numbering) were shown to be of importance for ATP potency [15, 71, 75]. In addition, conserved aromatic residues Phe185/Thr186 (P2X1) and Asn290/Phe 291 (P2X1) of a conserved NFR motif were shown to contribute to agonist action [76] and proposed to be involved in the coordination of the adenine ring [77, 78]. Studies using P2X2, P2X3, or P2X4 receptors revealed that conserved corresponding residues are responsible for ATP binding in these P2X receptors [69–74]. However, non-conserved amino acid residues contribute to the heterogeneity in pharmacological properties and play an equally important role to conserved residues in defining P2X receptor function [16, 78–82].

In a disulfide cross-linking study, it was shown that coexpressed P2X1 K68C and F291C mutants form an intersubunit cross-link in the absence but not in the presence of ATP, indicating that the ATP binding site is located at the interface of two adjacent subunits [83]. This is in line with functional studies on binding site mutants in the P2X2/3 heteromer, which suggested that residues from different subunits interact in agonist binding [64]. All these findings are in good agreement with the position of the relevant amino acids in the crystal structure of the zP2X4 receptor. Based on this structure, it appears that the ATP binding site is formed by deep intersubunit grooves, which are 45 Å away from the TM domains and surrounded by the conserved residues implicated in ATP binding. These residues are provided by the “body domain” and the “left flipper” of one subunit and the “dorsal fin” of the neighboring subunit (Fig. 2). The Cys-rich “head” domain of the first subunit projects over this binding site [48]. For recent reviews, see Coddou et al., Evans, and Browne et al. [12, 16, 84].

However, the crystal was obtained in the absence of ATP, and therefore, its exact mode of binding is unknown. Likewise, the conformational changes governing channel opening and desensitization remain elusive. The proposed position of the ATP binding site was supported by cysteine scanning mutagenesis and homology modelling using the zP2X4 crystal structure as template for a P2X1 homology model [85]. Its localization was further corroborated by a functional study showing that the thiol-reactive ATP-analogue NCS-ATP can be covalently attached to introduced cysteine residues (N140C or L186C) located at two adjacent subunits within the proposed ATP binding cavity in the P2X2 receptor [86]. Interestingly, covalent attachment of NCS-ATP to these introduced cysteines resulted in agonist-bound states that differ in the ability to gate the channel, suggesting the existence of at least two binding modes of ATP and allowing speculations on the reaction scheme of P2X ligand binding and opening [86].

#### Antagonist binding

Although selective competitive P2X receptor antagonists, like NF449 or A-317491 are known, studies dealing with the molecular action of these antagonists are infrequent. Instead, several studies of antagonist binding are performed with the non-selective negatively charged antagonists PPADS [16, 87–90] and suramin [75, 76, 90, 91]. As several reviews describing these data are available [12, 16, 84], we will focus here on the recent findings on the molecular action of the P2X1 receptor selective antagonist NF449 and the P2X2 receptor antagonist NF770 which were obtained by mutagenesis combined with docking studies on homology models based on the zP2X4 receptor structure.

A study on the antagonistic action of NF449 and suramin at the P2X1 receptor [92] identified a cluster of positively charged residues (^136^KAKRK^140^) at the base of the Cys-rich head domain that is responsible for the P2X1-selective antagonism of NF449 and absent in P2X2 receptors. However, these residues are not exclusive determinants of the selective antagonism by NF449, since reciprocal mutations in the P2X2 receptor only modestly increased the NF449 sensitivity, suggesting a more complex interaction with other non-conserved residues [92]. Nevertheless, this study highlights the importance of the Cys-rich region for normal channel function and ligand binding at human P2X1 receptors [92], as already proposed from studies on the *D. discoideum* P2X receptor that lacks this region [27, 30].

**Fig. 3:**
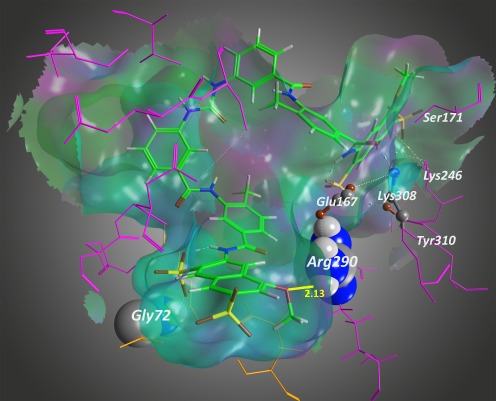
Proposed binding of the antagonist NF770 to the P2X2 receptor. The suramin derivative NF770 (7,7-(carbonylbis(imino-3,1-phenylenecarbonylimino-3,1-(4-methylphenylene)carbonylimino))bis(1-methoxy-naphthalene-3,6-disulfonic acid) tetrasodium salt)) is shown within the rP2X2 receptor binding pocket. Selected residues of the rP2X2 receptor binding site are shown as pink sticks, side chains of Gly72, Arg290, Glu167 and Lys308 are shown as ball and stick or space filling. NF770 is directed by a Gly72-sulfonate group (yellow/brown sticks) interaction to orient spatially in a way that the methoxy group oxygen (brown stick) comes into close apposition to Arg290. This way, a hydrogen bond can form that is a key determinant of the interaction of NF770 with the rP2X2 receptor. The close distance of 2.13 Å between the methoxy group and Arg290 (yellow bar) appears to account for the strong binding. The rP2X2 receptor homology model based on the X-ray structure of zP2X4.1 was generated by MOE2008.10. The receptor model was kept rigid during the docking computation, whereas the NF770 was allowed to remain flexible. For further details, see Wolf et al. [93]. The figure was generated and kindly provided by Achim Kless, Grünenthal GmbH, Global Drug Discovery

Structure–activity relationship analysis of suramin derivatives and in silico docking studies using a P2X2 receptor homology model revealed that residues important for potent antagonism such as Arg290 or Gly72 are also important in ATP action at P2X2 receptors [93] (Fig. 3). Furthermore, this study highlights the role of strong ionic interactions, for example, between the acidic groups of suramin derivatives and positively charged amino acid residues (Lys71, Lys246, Lys279, and Arg290) in the ATP-binding site, as suggested for the interaction with the phosphate oxygens of ATP [16, 64, 72, 84].

Thus, several additional and subtype-specific amino acid residues have been identified that play an important role for ligand selectivity and contribute to a comprehensive mapping of the orthosteric ligand binding site. This knowledge certainly will facilitate future ligand optimization by means of homology-model-based docking computation.

P2X receptors are also modulated by a variety of compounds including divalent cations, protons, lipids, steroids, ethanol, and ivermectin. As these allosteric modulatory sites of P2X receptors have been excellently reviewed recently [11, 12], they are not further considered here.

### Ion permeation pathway and channel opening

Several cysteine scanning mutagenesis studies suggested that TM2 lines the central ion-conducting pore and includes the channel gate at Thr336 (P2X2 numbering) and that TM1 is positioned peripheral to TM2 [94–98]. This spatial arrangement of the two TM domains was confirmed by the zP2X4 structure [48] and is quite similar to that found in the ASIC1 channel [49]. The TM helices within a subunit are oriented antiparallel to one another and are angled ~45° from the membrane plane with the inner TM2 helices defining most of the ion conducting pathway. They are surrounded by the peripheral TM1 helices, which make most of the contacts with the lipid bilayer [48, 49]. Along the threefold axis of symmetry of the P2X4 crystal structure, Kawate et al. identified four cavities, three in the ectodomain (upper-, central- and extracellular vestibules) and one located intracellularly (intracellular vestibule).

Based on the X-ray structure, the ion channel gate is presumably formed by residues Leu340 and Asn341 (corresponding to Ile332 and Asn333 in P2X2) on the extracellular side and by Leu346 and Ala347 (corresponding to Leu338 and Thr339 in P2X2) on the intracellular side of TM2. The closest association of the TM2 helices is Ala344, representing the center of the gate [48]. However, the weak side-chain density of the X-ray structure of the cytoplasmic terminus of TM2 complicates the side chain localization within the zP2X4 channel model. In subsequent studies, the pore and the gate of the P2X2 receptor were independently mapped, and potential opening movements were proposed [99–101]. Systematic mutagenesis of charged TM2 residues in combination with single channel analysis revealed that the side chains of Asn333, Thr336, and Ser340 (P2X2 numbering) are exposed to the permeation pathway within the open channel. It was proposed that the gate is formed by amino acid residues Asn333 to Thr339 and that the TM2 helices undergo a rotation and separation during channel opening [99]. Likewise, Keceli and Kubo found that the TM2 residue Thr339 orients towards the center of the permeation pathway. In addition, they provided evidence that residues Tyr43, Phe44, and Tyr47 in TM1 are oriented toward the pore-forming TM2 and interact with Ile328, thus stabilizing the closed state of the channel. This interaction is released in a voltage-dependent manner during gating of the channel [100]. By substituted cysteine accessibility analysis with the rapidly reversible Cd^2+^, Kracun et al. identified residues Thr339, Val343, Asp349, and Leu353 of TM2 lining deeper parts of the pore in the open state [101]. The different position of the P2X2 channel gate to that proposed by Li et al. [102] may be due to the use of the Cd^2+^ as a thiol modifying agent which allows to probe the rates of modifications of introduced cysteines and which is also smaller than the previously used thiol reactive methanethiosulfonate (MTS) compounds.

The question of how ions access the TM region of the channel has also been addressed. The zP2X4 structure suggests two pathways by which extracellular ions could enter the extracellular vestibule that allows access to the TM ion channel region [48]. First, three lateral fenestrations above the TM domains might allow cations to access this region. Second, ions might pass along the threefold axis of symmetry through the conspicuous upper and central vestibule to enter the extracellular vestibule and the TM channel region [48]. By using homology models of the human P2X1, rat P2X2, or human P2X4 receptor, residues that line the central and the lateral pathway were substituted by cysteine residues, and their reactivity to thiol-reactive MTS reagents during current recordings was investigated [85, 103, 104]. These studies concluded that ions enter the channels via the lateral pathway. Chambers along the central pathway were proposed to have a regulatory function [103], and the equivalent spacing of the three lateral portals was suggested to split the ion flow and thus minimize ion diffusion [104].

As the zP2X4 structure was obtained in the absence of ATP, it most likely represents the closed resting state of the channel [48]. Without a structure of the open state, the conformational changes involved in channel opening are difficult to predict. The mutagenesis and modelling studies suggest a dilation of the gate by a rotation [99] of the TM2 helices or by intrahelical movements resulting in less bending and a steeper position within the lipid bilayer [101]. Isoform specific amino acid differences in the region of the gate may account for variations in the occlusion point [101]. The proposed rotation and sliding of the TM2 helices against each other that leads to their separation is consistent with the functional finding that P2X2 receptor channel opening is prevented when Ile 328 (at the outer end of TM2) is tethered by an engineered disulfide to Val48 in TM1 [105].

A more recent cysteine scanning mutagenesis study using Ag^+^, Cd^2+^, and MTS reagents of different size suggests that the internal region of TM2 must move a large distance towards the central axis of the pore during opening, indicating that the pore-forming TM2 helices straighten from the steeply angled orientation toward the normal of the bilayer to open the channel [106]. According to the current view, ligand binding may be transduced into channel opening (resulting from TM movements) through the two structurally rigid β-sheets of the body domain (‘connecting rods’) of each subunit rising from the TM1 and TM2 [84]. The polar residues Glu63 and Arg274 localized within β-sheets β1 and β12, respectively, were identified to form an intersubunit salt-bridge that is likely to stabilize the closed state of the P2X2 channel [107]. Disulfide-bridge formation between the substituted cysteines E63C and R274C was reduced in the presence of ATP, suggesting that ATP binding might trigger relative movements of adjacent subunits at the level of Glu63 and Arg274, allowing the TM helices to open the channel [107]. Since these residues are not conserved in other subunits, subtype-specific mechanisms might govern channel opening.

### Channel desensitization

Based on whole cell recordings of heterologously expressed proteins, P2X receptors can be divided in rapidly desensitizing (P2X1 and P2X3) and slowly desensitizing (P2X2, P2X4, P2X5, and P2X7) receptors [10, 19]. The extent of desensitization of specific P2X receptors is of great physiological relevance as it determines the time course of P2X receptor signal transduction and regulates the responsiveness in the sustained presence of ATP. Desensitization represents the transition into a, most likely, agonist-bound closed form. It is followed by the recovery process that requires agonist unbinding and a conformational change from the agonist-free desensitized to the resting state [20]. The fact that the presence of high-affinity binding sites for ATP, α,β-meATP, and TNP-ATP appears to be associated with fast desensitization, and slow recovery from desensitization is in agreement with studies showing that the P2X1 receptor needs to open before it can go into the desensitized state, and that unbinding of the agonist from the ligand-bound desensitized state is the rate-limiting step for recovery from desensitization [20, 108, 109]. Consistent with these data, unbinding of [^32^P]-ATP from P2X3 receptors mirrored the rate of recovery from desensitization [20, 110].

The molecular mechanisms controlling desensitization are not yet understood and apparently involve multiple receptor segments (intracellular, TM, and extracellular domains) and possibly also interactions with other proteins or intracellular messengers [20, 37, 111]. Chimeras composed of parts of desensitizing and non-desensitizing P2X receptors indicate the involvement of the N-terminal- and TM1 domains [109, 112, 113] and a short N-terminal part of the ectodomain [114]. A recent study showed that substitution of solely intracellular N- or C-terminal parts of desensitizing P2X receptors by corresponding parts of non-desensitizing receptors is sufficient to obtain at least partial desensitizing or non-desensitizing receptors [37]. As shown by analysis of P2X2 splice variants and subsequent mutagenesis data, intracellular C-terminal parts next to TM2 contribute to the rate of desensitization [115, 116]. In the hP2X4 receptor, two residues in the proximal end of the C terminus, Lys373 and Tyr374, were found to accelerate desensitization [117]. Furthermore, it was shown that the positive charges of Lys365 and Lys369 within the C-terminal domain of P2X2 receptors are responsible for the interaction with membrane phosphoinositides and regulate desensitization of P2X2 receptors [118]. The extent of C-terminal controlled desensitization was suggested to influence the efficacy of the agonists [119]. Interestingly, the study of Bavan et al. showed that the penultimate C-terminal charge of the arginine residue of the BmP2X receptor is responsible for the slow desensitization kinetics but not the current run-down during repetitive ATP applications, indicating that run-down and desensitization are governed by distinct mechanisms [37].

In addition to the above described contribution of the intracellular C-terminal part, disruption of the putative conserved PKC phosphorylation site (see also section on “Phosphorylation of P2X receptors”) in the N terminus (^18^TXK^20^) by T18A or K20T mutations led to fast desensitization of P2X2 receptors [41, 121]. In contrast, the K20C mutation did not affect the P2X2 desensitization kinetics, indicating that this is a structural rather than charge effect [105]. The corresponding mutations in the P2X1 or P2X3 receptors result in rudimentary functional or non-functional receptors, respectively [41, 121, 122]. In support of the involvement of cytosolic components, inactivation properties of P2X2 receptors have been shown to differ greatly between measurements in excised patches and in whole cell mode [123].

In conclusion, these data suggest that desensitization is determined mostly, but not exclusively by the N- and C-terminal P2X receptor segments. Since the crystal structure of the zP2X4 is lacking these intracellular termini, we cannot infer their possible structural involvement in these processes.

## Pharmacological characteristics of P2X receptors

### Agonists

The primary agonist of all homomeric and heteromeric P2X receptors is ATP. Regardless of the species-dependent differences, the amount of ATP necessary to elicit the half maximal response (EC_50_) varies between sub-micromolar concentrations for P2X1, P2X3, and P2X5, and low micromolar concentrations for P2X2, P2X4, and P2X6 receptors [7, 124]. The P2X7 receptor requires exceptionally high agonist concentrations with an EC_50_ value for ATP higher than 100 μM [79, 125]. Remarkably, at this receptor, ATP is only a partial agonist. Extracellular Mg^2+^ ions diminish the agonist response at P2X7 receptors, an observation that has been interpreted as ATP^4-^ being the active agonist. Further experiments are needed to define whether this applies to other members of the family and to clarify to which extent divalent cations act directly at the receptor as negative modulators. In the absence of extracellular Mg^2+^ and other divalent cations, hP2X7 receptors were shown to have high and low affinity sites for free ATP^4-^ with apparent dissociation constants of 4 and 220 μM, respectively [126]. In addition to ATP, most P2X receptors are activated by diadenosine polyphosphates or related dinucleotides and some nucleoside triphosphates such as CTP and GTP [7, 12]. By contrast, the breakdown products of ATP, ADP, AMP, adenosine, or UTP and UDP activate P2X receptors either weakly or not, further corroborating the importance of the interaction with the three phosphate groups [12].

Early pharmacological studies have used the non-hydrolyzable ATP analogue α,β-meATP to differentiate between fast and slowly or non-desensitizing P2X receptors in smooth muscle and sensory neurons [4]. After cloning and heterologous expression of the seven subtypes, the P2X1 and P2X3 receptors were found to be sensitive to α,β-meATP (EC_50_ ≤ 1 μM), [17, 18, 66]. Heteromeric assemblies, which contain P2X1 or P2X3 subunits and heteromeric P2X4/6 receptors also show α,β-meATP-sensitivity (EC_50_ ≤ 10 μM) [17, 66]. At P2X7 receptors, 2’-3’-*O*-(4-benzoylbenzoyl)-adenosine 5’-triphosphate (BzATP) is a more potent agonist than ATP (EC_50_ ~10 μM) [79, 125]. In addition, BzATP activates particularly P2X1, P2X2, and P2X3 receptors with high potency [17, 127]. Furthermore commonly used ATP derivatives are ATPγS, which activates all P2X receptors with exception of the P2X7 receptor, and 2-MeS-ATP, which activates most P2 receptors but not adenosine (P1) receptors [7, 12].

A further peculiarity exists for the P2X7 receptor: For the mouse P2X7 receptor, it was shown that it can be activated by low concentrations of extracellular NAD. This process involves ADP-ribosylation of the P2X7 Arg125 by ecto-ADP-ribosyltransferase and results in constitutive channel activation [128, 129].

### Antagonists

Research in the P2X field has for a long time been hampered by the unavailability of truly selective antagonists [66]. P2X receptors have attracted widespread interest as therapeutic targets, e.g., for chronic inflammatory diseases and pain. In particular, P2X3 and P2X7 receptor antagonists have been developed and demonstrated antinociceptive or antiinflammatory effects in animal models of these diseases [17, 130]. In basic research, a variety of more or less selective compounds and their derivatives have been used, including dyes (e.g., phenol red, reactive red, reactive blue II, trypan blue, Evans blue, and brilliant blue), the antitrypanocidal drug suramin, the photoreactive agent ANAPP3, the cross-linking reagent DIDS, and the pyridoxal-5-phosphate analogue PPADS [7, 12, 17, 66, 127, 131]. In addition, trinitrophenyl-substituted nucleotides, especially TNP-ATP, are potent antagonists at P2X1, P2X3, and heteromeric P2X2/3 receptors [132]. A considerable additional problem are species-dependent differences in the action of both agonists and antagonists at P2X receptors (reviewed in Gever et al. and Donnelly-Roberts et al. [66, 79]). For example, the isoquinoline compounds KN-62 and KN-04 antagonize mouse [133] and human [134] P2X7 receptors but are inactive at the rat isoform [135].

Since the available P2X receptor antagonists have been extensively reviewed [7, 12, 17, 66, 127], we will focus here on more recently described and novel compounds that give new insights in ligand binding of P2X receptors and/or have proven to be useful tools in animal models of diseases or even progressed into clinical studies in man.

The potential of suramin as a lead structure for development of selective and/or potent P2X receptor antagonists has been shown in several studies [93, 136–138]. Its derivative NF449 is currently the most potent and highly selective P2X1 receptor antagonist (EC_50_ < 1 nM) [137, 139]. Together with suramin related compounds, such as NF770, which is a potent but less selective P2X2 receptor antagonist, it helped to understand competitive action of suramin and the basis of their subtype specificity [92, 93] (see section “Ligand binding sites”). Evaluation and optimization of anthraquinone derivatives related to reactive blue 2 yielded the first potent and selective P2X2 receptor antagonist PSB-1011 [140].

The first selective and highly potent dual inhibitor of P2X3 and P2X2/3 receptors, A-317491, showed strong antinociceptive effects in vivo in rodent models of chronic inflammatory and neuropathic pain [141], thus providing an important proof of concept. It was not pursued as a drug candidate due to its poor distribution into the central nervous system [66, 142]. Selective dual inhibition of P2X3 and P2X2/3 receptors has also been achieved by the nanomolar potent and orally bioavailable diaminopyrimidine derivatives RO-3, RO-4 (recently redesignated AF-353), and RO-51 developed at Roche [17, 142–144]. In particular, AF-353 (previously known as RO-4) was shown to bear a favorable pharmacokinetic profile and excellent antagonist potency and selectivity for P2X3 and P2X2/3 receptors [142]. Furthermore, it was able to attenuate bone cancer pain behavior in rats [145]. RO-85, an orally bioavailable drug-like P2X3 receptor antagonist, is selective for the P2X3 receptor over the P2X2/3 and other P2X receptor subtypes [146]. Three additional P2X3 and P2X2/3 selective diaminopyrimidine derivatives (compounds A, B, and C) with nanomolar potency were recently published by GlaxoSmithKline [147]. Compound B was shown to exhibit significant effects in the CFA model of chronic inflammatory pain [147]. AF-219 is another P2X3 receptor antagonist and the lead compound of Afferent Pharmaceuticals. It has successfully completed two phase-I clinical studies and progressed into phase II clinical testing (personal communication and see release at www.afferentpharma.com, accessed Aug. 22nd, 2011).

5-BDBD (5-(3-Bromophenyl)-1,3-dihydro-2 H-benzofuro-[3,2-e]-1,4-diazepin-2-one) was developed by Bayer Healthcare as a P2X4 receptor antagonist for treatment of arteriosclerosis and restenosis [148]. However, its impact is still unclear.

The strong evidence for P2X7 receptor involvement in pain and inflammation boosted interest in the pharmacology of this receptor, and selective P2X7 receptor antagonists have been frequently discovered in the past few years [17, 149]. Selectivity and in vitro activity at heterologously expressed human (and partly rat) P2X7 receptors has been shown for A-804598 and further compounds from Abbott [150–152], AZ11645373 [153]; several compounds from GlaxoSmithKline [154–156]; and compounds from Pfizer [157]. The newly developed nanomolar potent P2X7 receptor-selective antagonists GSK314181A (and further GSK compounds), A-740003, A-438079, and A-839977 have in addition been shown to have in vivo analgesic effects in rodent models of inflammatory pain [158–164]. The AstraZeneca compound AZD9056 was the first P2X7 receptor antagonist that entered clinical trials and was well tolerated in phase I studies [165]. Unexpectedly, it failed to show significant efficacy in the treatment of rheumatoid arthritis in a phase IIb clinical study, suggesting that the P2X7 receptor is not a therapeutically useful target in rheumatoid arthritis [166]. It is currently in clinical testing for the treatment of osteoarthritis, chronic obstructive pulmonary disease, and inflammatory bowel disease [165]. Likewise, CE-224535, a P2X7 receptor antagonist from Pfizer was reported to have no effect in phase II studies for the treatment of rheumatoid arthritis and is now studied for treatment of other conditions, such as pain and Alzheimer´s disease [167]. Furthermore, the P2X7 receptor antagonist GSK1482160 from GlaxoSmithKline has recently finished phase I clinical testing (ClinicalTrials.gov identifier: NCT00849134). The P2X7 receptor antagonist EVT-401 from Evotec is under development in the companion animal market for the treatment of inflammatory conditions (http://www.evotec.com, accessed 22 Aug 2011).

Also, currently approved drugs have been identified that act on P2X receptors. For instance, aminoglycoside antibiotics have been shown to block P2X2 receptor channels [168]. Several antidepressants, in particular, paroxetine, were shown to inhibit ATP-evoked rat and human P2X4 receptor-mediated responses [169]. Furthermore, intrathecally injected paroxetine produced significant antiallodynic effects in a rat model of neuropathic pain. In contrast, the tricyclic antidepressant amitriptyline exhibited only weak or no P2X4 inhibitory activity [169, 170]. However, a recent study indicates that the antidepressants act indirectly by interfering with P2X4 receptor trafficking [171] rather than directly blocking the receptors. Lately, the approved H_1_ antihistaminic clemastine was shown to act as a positive modulator of the P2X7 receptor [172].

## Synthesis and trafficking of P2X receptors

Cellular activity depends on the interaction between membrane receptors and intracellular signalling pathways and is critically regulated by the spatial and temporal distribution of the membrane receptors. For example, the control of receptor function by protein biogenesis, trafficking, and redistribution represents a central process in synaptic plasticity.

### P2X receptor synthesis

The appearance of functional ion channels in the plasma membrane follows a complex series of events, including specific oligomerization of protein subunits as well as post-translational folding and modification. Like other membrane proteins, P2X receptors are synthesized and core glycosylated in the rough ER and, upon complex glycosylation in the Golgi apparatus, are trafficked to the plasma membrane in a constitutive or regulated pathway of vesicle exocytosis [173]. In both cases, specific vesicle docking to target membranes is achieved by distinct members of the SNARE (soluble *N*-ethylmaleimide-sensitive factor (NSF) attachment protein receptors) protein family. This process is counterbalanced by a clathrin-mediated endocytosis of receptors to the endosome where they are further sorted into vesicles, depending on their final destination (degradation or recycling). P2X polypeptides assemble quickly into trimers since monomeric subunits or intermediate dimeric assembly states were never observed with metabolically labelled P2X1 protein expressed in oocytes [44]. Besides formation of disulfide bonds [40, 68], post-translational modification by *N*-linked glycosylation is important for delivery of functional channels to the plasma membrane. The seven rat P2X subunits contain three to six consensus sequences for *N*-linked glycosylation in their extracellular domain. Systematic mutagenesis studies at the P2X1 [174], P2X2 [175], P2X3 [176], and P2X7 receptors [177] suggest that a minimum number of two *N*-glycans is essential for efficient plasma membrane targeting. The glycosylation site equivalent to Asn 170 in P2X3 is the best conserved among the P2X subtypes and appears to play also a critical role for receptor function [71, 77, 176, 177], which might be explained by its close location to the proposed ATP binding site.

### P2X receptor trafficking

A YXXXK motif in the C terminus is common to all P2X subunits. It is located downstream of the second TM domain, except for the P2X7 subunit where a cysteine-rich domain of 18 amino acids lies between the second TM domain and this motif [178]. The YXXXK motif regulates the surface expression of P2X receptors, and accordingly, its mutation significantly limits the trafficking of homomeric P2X receptors. Trafficking of mutant subunits is rescued by heteromerization with wild-type subunits. The YXXXK motif is proposed to stabilize membrane inserted P2X receptors, rather than affect ER exit [178]. Unstable mutants are rapidly internalized and directed to the lysosomal pathway for destruction. The expression and plasma membrane transport of P2X receptors is highly regulated by cell activity and maturation. However, the trafficking mechanisms for individual P2X receptors are poorly understood, and hardly any interacting proteins controlling these processes have been identified so far.

### P2X1 receptors

P2X1 receptors show fast desensitization and long recovery periods until full reactivation is possible. The desensitization and recovery appears to be determined by two mechanisms: first, intrinsic receptor properties leading to fast conformational changes upon ATP binding and slow unbinding of ATP allowing a delayed return to the resting state. In addition, agonist-induced internalization and redistribution of receptors between plasma membrane and intracellular compartments has been described for heterologously expressed GFP-tagged P2X1 receptors [179, 180] and native P2X1 receptors in smooth muscle [181]. A recent fluorescence recovery after photo-bleaching (FRAP) study suggests that both a constitutive brefeldin A-sensitive and an agonist-induced dynasore-sensitive trafficking pathway contribute to the recycling of P2X1 receptors [182].

### P2X2 receptors

Agonist-induced receptor clustering associated with increased current responses and dendritic morphology changes, but no net internalization or externalization has been demonstrated for GFP-tagged P2X2 receptor expressed in embryonic hippocampal neurons [183]. Interestingly, this effect was not seen if the PKC consensus site of P2X2 was disrupted by a T18A mutation. Supporting evidence for agonist-induced clustering of P2X2 receptors is provided in a recent study on spinal cord neurons [184]. Here, a proportion of P2X2 receptors appear to directly interact with and stabilize GABA_A_ receptors, which in turn help their trafficking to extrasynaptic localizations in the plasma membrane.

### P2X3 receptors

Expression of P2X2 and the fast desensitizing P2X3 receptors is upregulated in DRG neurons from rats with peripheral inflammation [185], resulting in increased ATP-responses and sensitization of the neurons to ATP. An increase in plasma membrane trafficking of P2X3 receptors was found to be responsible for this effect [186]. Upon electrical stimulation to mimic the injurious state, CaMKII is likewise upregulated and has been shown to promote trafficking of P2X3 receptor in the plasma membrane [187]. A recent study on transfected HEK cells and primary cultures of DRG neurons found that the P2X3 receptor undergoes rapid constitutive endocytosis and is predominantly localized in intracellular compartments labelled by the late endosome/lysosome marker lamp1. Upon agonist application, the level of functional receptors in the plasma membrane is rapidly upregulated [188]. In trigeminal neurons, the trafficking to the plasma membrane and activity of P2X3 receptors was shown to be regulated by calcitonin-gene-related peptide and nerve growth factor via PKA and PKC, respectively [189].

### P2X4 receptors

Trafficking processes have been best characterized for the P2X4 receptor. Upregulation of P2X4 receptors in spinal microglia, as a result of peripheral nerve injury, has been shown to be an important determinant of neuropathic pain [190, 191]. If heterologously expressed in neurons, this receptor undergoes rapid constitutive- and agonist-induced internalization into early endosomes and lysosomes from where they are subsequently reinserted into the plasma membrane [192]. Internalization of the P2X4 receptor is clathrin- and dynamin-dependent and determined by a non-canonical endocytic motif (YXXGL) downstream of the conserved YXXXK motif and a canonical YXXV motif. This YXXGL motif was shown to interact with adapter protein 2 (AP2), and mutation of this endocytic motif or the Tyr binding pocket in the μ2 subunit of the AP2 clathrin adaptor protein complex resulted in accumulation of functional P2X4 receptors in the membrane [193]. An intact endocytosis motif also appears to be required for the enhancement of P2X4 receptor currents by protein kinase A, suggesting that the endocytotic pathway is regulated by phosphorylation [194]. Similarly, impairment of P2X4-endocytosis by the positive modulator ivermectin has been suggested as one mechanism underlying enhancement of P2X4 receptor responses [195]. However, this mechanism is controversially discussed [196].

In addition to the non-canonical endocytic motif, a N-terminal dileucine-type motif appears to contribute to lysosomal targeting of P2X4 receptors [197]. Native P2X4 receptors in microglia, macrophages, and endothelial cells are localized primarily in lysosomes where their *N*-glycans protect them from degradation. Stimulation of lysosome exocytosis by ionomycin-induced rise in intracellular Ca^2+^ concentration or methylamine-induced rise in lysosomal pH enhanced P2X4 expression and responses at the plasma membrane. This suggests that the lysosomal pool of P2X4 receptors can be mobilized to upregulate P2X4 responsiveness of these cells [197]. Likewise, lysosome-localized P2X4 receptors were inserted into the plasma membrane in lipopolysaccaride (LPS)-activated C8-B4 microglia, a cell line of cerebellar origin. Interestingly, this lysosomal secretion was reduced by clinically relevant concentrations of antidepressants, providing a possible explanation for their effectiveness in neuropathic pain models [171]. In contrast, activation of human alveolar or rodent macrophages by IFN-γ plus LPS or TNF-α resulted in decreased P2X4 responses while induction of lysosomal secretion (pH increase of intracellular vesicles by chloroquin) or phagocytosis (ingestion of zymosan particles) increased the appearance of functional P2X4 receptors in the plasma membrane [198]. Also in bone-marrow-derived macrophages, large amounts of P2X4 receptors were predominantly localized intracellularly, and treatment with the endocytosis inhibitor dynasore did not enhance surface expression, suggesting a much less dynamic trafficking than in microglia [52].

The predominant intracellular localization of some P2X receptors, in particular, the P2X4 subtype, makes it tempting to speculate about intracellular functions of P2X receptors in certain cell types. In simple eukaryotes, for example, P2X receptors localized in intracellular membranes were shown to be involved in osmoregulation [27] even though playing only a minor role [30]. It remains to be determined whether mammalian P2X receptors can also fulfil specific intracellular roles [52, 199].

### P2X5 and P2X6 receptors

Recombinant rodent and zebrafish P2X5 receptors show low current responses despite good expression on the protein level. In contrast, chick and bullfrog P2X5 receptors show good functional expression [57, 200–202]. The reason for this is not clear. The human P2X5 receptor is non-functional due to the deletion of exon 10 but gives good responses if the missing sequence is included, as in some individuals that carry a polymorphism in which the critical intronic splice site is preserved [55].

Heterologous expression of functional rat P2X6 receptors has been reported in only a low percentage of HEK cells [200, 203] and not at all or only at the detection limit in *Xenopus* oocytes [204, 205]. The mouse and human isoforms of P2X6 also do not express well [206]. Biochemical analysis revealed that the majority of heterologously expressed P2X6 subunits failed to form trimers and were retained in the ER [45, 53, 54]. Homotrimeric assembly and trafficking to the plasma membrane could be enhanced by mutation or deletion of an uncharged region in the P2X6 N terminus [207]. Differential glycosylation of P2X6 subunits in HEK cells has also been proposed to account for inconsistencies in the functional expression of this subtype [203].

### P2X7 receptors

Together with P2X4 receptors, P2X7 receptors are predominantly expressed in endothelial and epithelial cells, and cells of the immune system where the level of functional P2X7 receptors in the plasma membrane is also tightly regulated. In monocytes and lymphocytes, for example, they are localized intracellularly and appear to be recruited to the plasma membrane during differentiation of monocytes into macrophages [208–210]. In macrophages and microglia, they appear predominantly at the cell surface [52]. Two basic amino acid residues (Arg578, Lys579 [211]) within an LPS-binding motif (residues 573–590 [212]) have been shown to be critical for efficient surface localization, presumably by stabilizing the receptor in the plasma membrane. Likewise, truncations or mutations (residues C572G, R574G, F581G) in an overlapping region between residues 551 and 581 [213] in the P2X7 C terminus abolished surface expression, and it was suggested that they contain an ER retention/retrieval motif. In agreement with the importance of this region, the I568N polymorphism in this domain [214] causes deficits in surface expression. In rat submandibular gland, a fraction of P2X7 receptors was found in lipid rafts [215]. Also in alveolar epithelial cells, P2X7 receptors were found to co-localize with caveolin-1, and deletion or suppression of this protein resulted in a strong reduction of P2X7 immunoreactivity [216]. A study on transfected HEK cells and macrophages [217] found that palmitoylation is involved in the correct targeting of P2X7 receptors into lipid rafts and correlates with its plasma membrane expression. While more distal groups of cysteine residues (Cys477, Cys479, Cys482/Cys498, Cys499, Cys506/Cys572, Cys573) are essential, juxtamembrane cysteine residues (Cys371, Cys373, Cys374) also appear to be involved in palmitoylation. Interestingly, the essential residues include Cys572 and Cys573, which are located in the above-mentioned regions [211–213]. Palmitoylation-deficient mutants were retained in the ER, and it was concluded that palmitoylation is required for P2X7 receptor maturation. Agonist-induced down regulation of P2X7 receptors was shown in RAW macrophage-like cells [218].

## Regulation and protein interactions of P2X receptors

Apart from yet unidentified subunit combinations or splice variants, transient or permanent physical interactions with associated proteins can account for diversity in P2X receptor properties. Transiently associated proteins include proteins involved in protein synthesis and maturation, such as enzymes involved in glycosylation and chaperones, as well as proteins that participate in the trafficking and stabilization of the receptor at specific membranes, such as adaptor, anchoring, and scaffolding proteins (see section “Synthesis and trafficking of P2X receptors”). In addition, the functions of a mature receptor can be modified by intracellular signalling molecules, by enzymes, such as kinases, and by cross-talk with other receptors or membrane proteins.

### Phosphorylation of P2X receptors

In addition to several *N*-linked glycosylation sites, P2X receptors contain a conserved putative PKC phosphorylation site (Thr-X-Arg/Lys). Disruption of this N-terminal PKC site in the P2X1 receptors alters the time course of desensitization, suggesting that desensitization is regulated by phosphorylation [122]. Indeed, basal P2X1 receptor phosphorylation was demonstrated by [^32^P]orthophosphate labelling in HEK293 cells expressing this receptor. However, the effect on desensitization was found to be indirect and rather involves phosphorylation of an accessory protein [219]. Potentiation of P2X1 receptor-mediated responses by the PKC activator phorbol 12-myristate 13-acetate (PMA) or stimulation of coexpressed mGluR1α receptors was abolished after disruption of the N-terminal phosphorylation motif or by mutations within the C-terminal region between His355-Tyr370, indicating regulatory roles of both the N-terminal and C-terminal domains [220].

In *Xenopus* oocyte-expressed P2X2 receptors, the phosphorylation motif likewise controls the desensitization kinetics and phosphorylation of Thr18 was demonstrated with a phosphothreonine–proline-specific antibody [41]. In contrast, direct phosphorylation of P2X2 receptors expressed in *Xenopus* oocytes or HEK293 cells could neither be detected by immuno-blotting nor by in vitro and in vivo phosphorylation assays in another study [121]. Nevertheless, the role of the ^18^ThrProLys^20^ motif for desensitization kinetics was confirmed. Three studies on P2X3 receptors have shown that the PKC activator PMA increases P2X3 receptor-mediated current amplitudes, but, in contrast to the findings with P2X1 receptors, no phosphorylation was detected [121, 221, 222]. Interestingly, a PKC consensus site in the P2X3 ectodomain was shown to be regulated by ecto-PKC, resulting in changes of the αβ-meATP-induced current responses [223, 224].

A cAMP-dependent protein kinase A-mediated regulation of the P2X4 receptor function via C-terminal motifs was also shown [194]. For P2X7 receptors, it was reported that receptor activation results in dephosphorylation of Tyr343 within the second TM domain, suggesting its basal phosphorylation [225].

### Clustering of P2X receptors and interactions with ion channels

Several studies provide functional evidence for interactions between homotrimeric P2X2 receptors. Properties such as mean open times, open channel noise [226], potentiation by Zn^2+^, and pH, as well as the EC_50_ value for ATP appear to depend on receptor density [227]. Also, the ability to form large pores and inward rectification properties were shown to depend on the P2X2 expression level [228] and to be influenced by mutation of amino acid residue Ile328 in the second TM domain. A physical interaction between P2X2 receptors can be inferred from biochemical experiments that show an increased tendency of this receptor to form higher-order complexes [54]. Functional and physical interactions between P2X7 and P2X4 [229–231] and recently also P2X2 and P2X4 receptors [232] have been observed, although heterotrimerization between these subunits was excluded [52, 232, 233]. Together, these data suggest that some P2X trimers can interact with each other either directly or via clustering molecules. Whether these interactions have physiological relevance or represent overexpression artifacts remains to be determined. Interestingly, P2X4 and P2X7 could be coprecipitated with the extracellular matrix component biglycan and soluble biglycan-induced clustering of P2X4 and P2X7 receptors with Toll-like receptor (TLR) 2/4 was found to underlie the activation of the inflammasome by this component [234].

A wealth of functional and biochemical evidence exists for interactions between P2X receptors and various members of the Cys-loop superfamily of ligand-gated ion channels. Functional interactions resulting in cross-inhibition have been described in native and/or recombinant systems between P2X receptors and γ-aminobutyric acid receptors [235–238], nicotinic acetylcholine receptors [239–244], and 5-hydroxytryptamine receptors [245, 246]. There is evidence that the P2X2 receptor, via its C terminus, physically interacts with GABA_A_Rs and GABA_C_Rs [235, 238] and that co-transfection of P2X2 subunits modulates their targeting in transfected hippocampal neurons and spinal cord neurons [184]. Similarly, two (Tyr374, Val375) and three (Gln386-Thr388) amino acid residues in the P2X3 and P2X4 C termini, respectively, enable an inhibitory cross-talk with GABA_A_ receptors in DRG [247] and hypothalamic neurons and thus regulate synaptic transmission [248]. A detailed analysis of the interaction between P2X2 receptors and the α4β2 nAChR by FRET combined with total internal reflection fluorescence microscopy indicates that both channels are closely associated (approximately 80 Å apart), suggesting that they form functional dimers of two receptor complexes [249].

Coexpression of P2X subunits with ENaC resulted in mutual regulation of channel trafficking in *Xenopus* oocytes [250]. More recently, a close interaction between P2X receptors and another member of the amiloride-sensitive Na^+^ channel family, the acid-sensing ion channel (ASIC), was reported in sensory neurons [251]. According to this study, the electrically quiet P2X5 receptor forms a molecular complex with ASIC3 and increases its pH sensitivity, thereby forming a coincidence detector for low pH and ATP in muscle ischemia. Finally, a functional Ca^2+^-dependent interaction between P2X receptors and *N*-methyl-d-aspartate (NMDA) receptors has been described in hippocampal pyramidal neurons and appears to play a role in the modulation of synaptic plasticity [252, 253].

Much attention was raised by the finding that the P2X7 receptor and the hemichannel pannexin-1 could be co-purified from transfected HEK cells and that a functional interaction of both proteins was shown in different cell types. Based on these data, it was concluded that pannexin-1 constitutes the "P2X7 pore" and is required for processing of caspase-1 and subsequent release of mature IL-1β [254]. Other studies, however, could not confirm this finding [255–258], and the pore-forming mechanism remains to be elusive (see section “Non-specific pore formation”).

### Interactions of P2X receptors with other proteins

Due to their particular longer C termini that can be used as baits in "pull-down" assays, most interactions have been determined for P2X2 and P2X7 receptors. Using the C terminus of the P2X2 receptor as a bait in GST-pull-down or yeast two-hybrid assays, βIII tubulin, myelin basic protein [259], heat-shock protein 90 (HSP90), vacuolar-type H^+^-adenosine triphosphatase, NSF, tubulin 1α, vesicle amin transport protein 1 (VAT1), glutamic acid decarboxylase synapsin IIb, glutamine synthetase, visinin-like protein 1 (VILIP1) [260], as well as Fe65 and Fe65-like adaptor proteins were identified as associated proteins [261]. βIII tubulin was found to bind to a prolin-rich segment (371–412) in the P2X2 C terminus.

Fe65 is a brain-enriched multidomain adaptor protein containing one WW protein interaction domain and two phosphotyrosine binding/interacting domains (PTB/PID). It has been shown to interact with amyloid precursor proteins and to be involved in brain development [262]. Interaction with the P2X2 receptor appears through the WW domain and the alternatively spliced P2X2b that lacks a C-terminal segment of 69 amino acid residues was not able to interact with Fe65. Co-localization of the P2X2 receptor with Fe65 at postsynaptic specializations of excitatory synapses in the hippocampus was shown by immunogold labeling, and both proteins could be co-precipitated from rat brain extracts. Functional analysis showed that pore dilation of the P2X2 receptor (see section “Non-specific pore formation”) was inhibited upon co-expression of Fe65 [261].

VILIP-1 is a member of the neuronal EF-hand Ca^2+^-sensor protein family. It has been shown to interact also with a nicotinic ion channel and plays a role in regulating cAMP levels, cell signalling, and membrane trafficking [263]. P2X2 receptors and VILIP1 were co-localized in deep cerebellar nuclei, and the dentate gyrus and both proteins could be co-immunoprecipitated from brain extracts. Co-expression of VILIP1 lowered the ATP sensitivity of P2X2 receptors and increased its membrane expression, peak responses, and diffusion in the plasma membrane. Further analysis indicated that a constitutive interaction via the P2X2 juxtamembrane region of the C terminus exists and is increased in an activation- and Ca^2+^-dependent manner, which could constitute a molecular feedback mechanism [260].

The multiple P2X7 receptor functions appear to be particularly dependent on its C terminus and associated proteins but only comparatively limited information on its interaction with other proteins is available. In rat but not in human P2X7 receptors, a Ca^2+^-dependent facilitation of P2X7 receptor responses was identified, and calmodulin could be co-immunoprecipitated with agonist-stimulated P2X7 receptors. By mutagenesis, a novel calmodulin binding motif was identified [264, 265].

Upon immunoprecipitation of P2X7 receptors overexpressed in HEK cells, 11 proteins were co-isolated including cytoskeletal proteins (supervillin, β-actin, and α-actinin), chaperones (HSP70, HSC71, and HSP90), the integrin β2 subunit, the extracellular matrix protein laminin α3, the scaffolding protein MAGuK, and the signalling molecules PI4K and receptor phospho-tyrosine phosphatase β (RPTPβ) [225]. The interaction with HSP 90 was further characterized [266]. Using a HEK cell library in a yeast two-hybrid assay, the epithelial membrane protein 2 and related proteins were found to directly interact with the P2X7 receptor C terminus [267].

In a more recent immunopurification study, two non-muscle myosins, NMMHC-IIA and myosin VA, were isolated from monocytic THP-1 cells and P2X7-transfected HEK cells, respectively. In line with the above study, an interaction with protein-tyrosine phosphatase, β-actin, and heat-shock proteins was also found, and in addition, the ubiquitin ligaseRo52, InsP6 and PP-IP5 kinase 1, myosin regulatory light chain, nucleoprotein TRP, tubulin, and nucleoside diphosphate kinase B were identified [268]. P2X7 receptors were shown to co-localize with NMMHC-IIA in HEK cells and functional characterizations by flow cytometry suggest that agonist-induced dissociation of the receptor is required for pore formation while the intact complex is required for phagocytosis in transfected HEK cells, human monocytes, and mice macrophages [269].

For P2X4 and P2X6 receptors, an interaction with the endothelial cell-specific adhesion molecule VE-cadherin was found in human endothelial cells [270].

In a recent co-purification study, a close interaction of the P2X1 receptor with the actin cytoskeleton was found and shown to occur via the P2X1-amino-terminus. The interaction was suggested to contribute to a localized signalling environment in lipid rafts [271].

In addition to the above-described protein interactions, for which in many cases evidence for a physical interaction was provided, P2X receptors have also been shown to functionally interact with a range of other proteins including G-protein-coupled receptors. For further details, please refer to Koeles et al. [272].

Investigation of the tissue-specific subunit composition of native P2X receptors and their protein–protein interactions has partly been hampered by the lack of P2X antibodies that are specific and suitable for immunoprecipitation. Such studies appear highly important in view of the entirely unknown composition of P2X receptor complexes in neuronal membranes where neither their subunit composition nor their enrichment at synaptic sites has been analyzed in detail.

## P2X receptor signalling

### Ion flux

P2X receptors are essentially non-selective cation channels permeable to small monovalent and divalent cations. P2X receptor activation generally leads to a change in membrane potential initiating subsequent cellular events. For instance, P2X receptor-mediated changes of the membrane potential in neurons presynaptically modulate neurotransmitter release [273–277] or postsynaptically result in fast excitatory signalling [18, 278]. The involvement of P2X receptor-mediated currents in signalling processes of virtually all cells, tissues, and organs is extensively reviewed elsewhere [3, 18, 21, 279]. Nevertheless, beside the direct change of the membrane potential, a major physiological mechanism by which activated P2X receptors control cellular functions is elevation in intracellular calcium concentration ([Ca^2+^]_i_) both directly by Ca^2+^ permeation and indirectly by facilitation of voltage-gated Ca^2+^ channels [111, 280, 281]. The fractional Ca^2+^ currents of recombinant P2X receptor subtypes were systematically analyzed by Egan and Khakh and vary between 2.7% and 12.4% (P2X1, 12.4%; P2X2, 5.7%; P2X3, 2.7%; P2X4, 11.0%; P2X5, 4.5%; P2X7, 4.6%; P2X2/3, 3.5%; P2X2/6, 7.7%; P2X4/6, 11.3%), which for some receptors is larger than the Ca^2+^ permeability of acetylcholine-, serotonin-, or glutamate-gated channels [282]. In contrast to the highly Ca^2+^-permeable NMDA receptors, however, P2X receptors can mediate Ca^2+^ influx at resting or low membrane potentials when NMDA receptors are not active. The increase in [Ca^2+^]_i_ activates a broad range of second messenger systems and signalling cascades and can trigger manifold short- and long-term cellular events. For instance, P2X receptors participate in synaptic transmission in the hippocampus by providing a component of the excitatory input to CA1 pyramidal neurons, in which the activation of P2X receptors generates calcium influx that does not require cell depolarization [253]. Inhibition of P2X receptors on these CA1 pyramidal neurons facilitates the induction of long-term potentiation (LTP), indicating that P2X receptors act via calcium influx as a dynamic low-frequency filter within the hippocampus [252, 253].

### Non-specific pore formation

It is generally assumed that ionic selectivity is an invariant property of specific ion channels. Several examples exist, however, of channels that have dynamic selectivity filters. These include proton-gated channels, cardiac sodium channels, and some Kv channels [283]. More recently, the TRPV1 receptor has been shown to dilate into larger pores that are permeable to the large fluorescent dye YO-PRO-1 [284]. The physiological significance of these permeability changes remains elusive.

For the slowly desensitizing P2X2, P2X2/3, P2X4, and P2X7 receptors, the development of an additional permeability state which allows the passage of the large cation *N*-methyl-d-glucamine (NMDG) and fluorescence dyes such as the cationic propidium dye YO-PRO-1, and ethidium has also been observed upon repeated applications or in the continuous presence (~30 s) of agonist [283, 285, 286]. This permeability change can also be monitored by a change in the reversal potential if experiments are performed in extracellular NMDG, an organic cation that generally does not efficiently permeate ion channels but does so during ATP-activated channel dilation [283, 285–287]. The two permeability states are referred to as I_1_ (permeability to small cations) and I_2_ (permeability to larger cations) [283, 287]. In the P2X4 receptor, they could be separated by exchange of the conserved residue Gly347 in TM2: Mutation into a tyrosine residue resulted in channels that lacked the large permeability I_2_ state while mutation into a positively charged residue strongly reduced the I_1_ current [283]. In the P2X2 receptor, mutation of residues Asn333, Thr336, Leu338, and Gly342 (analogous to Gly347 in P2X4) in TM2 into alanine residues appeared to favor opening of the I_2_ permeability state [285]. In a systematic alanine scanning mutagenesis study, a total of ten residues in the two TM domains (Phe31, Arg34, Gln37, Lys53, Ile328, Ile332, Ser340, Gly342, Trp350, Leu352) were identified to perturb transition from the I_1_ to the NMDG permeable I_2_ state [288]. Of these, Ile328 had also been shown in a previous study to be critical for expression level-dependent changes of the P2X2 receptor permeation properties [228]. Taking the orientation of the previously defined selectivity filter (Thr336, Thr339, and Ser340) [282] and the constraint that Val48 and Ile328 are close to each other [63, 289] as a basis, the I_2_ state-specific hits were mapped onto helical wheel representations. In agreement with an effect on protein–protein interactions in the TM region, the residues were found at the interface of neighboring TM1 and TM2 domains of adjacent subunits [288], and it was proposed that the permeation pathway could dilate by helix tilting, rotation, or bending as assumed for other channels [290].

An additional mutagenesis study showed that the pore dilation occurs only in rat but not mouse P2X2 receptors and is dependent on specific residues in the C-terminal domain, suggesting that changes in the permeation pathway during opening to the I_2_ state require conformational changes in the C terminus [287]. Interestingly and in support of this finding, the I_2_ state appears to be inhibited by interaction of the P2X2 receptor C terminus with the beta-amyloid precursor protein-binding protein Fe65 [261]. In addition, channel activity and pore dilation appear to be regulated by the interaction of membrane-bound phosphoinositides with the proximal region of the P2X2 receptor C-terminal domain [118]. Cytosolic gating motions in the N- and C-terminal domains were also shown and further analyzed by FRET studies with fluorescent proteins and FlAsh-labelled receptors. These studies also revealed that the pore dilation is not dependent on pannexin-1, which was proposed to be involved in pore dilation of P2X7 receptors [255, 291]. Together, all these data suggest that, at least in the P2X2 receptor, the pore dilation is an intrinsic property of the receptor. Most recently, it was shown that colchicine inhibits pore dilation but not ATP-gated currents of P2X2 and P2X7 receptors in oocytes and macrophages [292].

The P2X7 receptor shows a permeability increase with similar kinetics as the P2X2 and P2X4 receptors [285, 286]. In addition, the sustained agonist application leads to cell lysis and apoptosis [293, 294]. These features have not been observed with P2X2 and P2X4 subtypes and require the C terminus while YO-PRO-1 uptake in P2X7 receptors is strongly reduced but not abolished if the C terminus is removed [125, 213]. It remains to be answered whether the dilation of the P2X7 ion channel [285] reflects a property common to the P2X2 and P2X4 receptors. Based on patch-clamp measurements in the cell-attached configuration, it has been suggested that P2X7 receptor-associated pore formation might require ancillary proteins (e.g., either hemi-channels, or the maitotoxin-associated pore [295]) whose activation is dependent on the production of diffusible second messengers such as Ca^2+^ or MAP kinases [296, 297]. A study on P2X7-transfected HEK cells showed that NMDG permeability measured by reversal potential shifts and YO-PRO-1 uptake measured by fluorescence intensity could be differentiated: NMDG permeability but not YO-PRO-1 uptake was inhibited by both normal sodium concentration in the extracellular medium or deletion of a cys-rich 18-amino acid segment in the juxtamembrane C-terminal region of the receptor [298]. From these data, it was concluded that the NMDG permeability is an intrinsic channel property while YO-PRO-1 uptake requires a distinct permeation pathway. In addition, another study found that both a cationic and an anionic dye permeation pathway were opened by P2X7 receptor activation [299]. Since blockade of pannexin hemichannels inhibited P2X7 receptor-associated dye uptake in HEK cells and macrophages while its overexpression resulted in increased dye uptake and both proteins were co-purified from transfected HEK cells, pannexin was suggested to interact with the P2X7 receptor and to be the cause of pore formation [254]. However, more recent data do not confirm this hypothesis [255–258]. In support of an NMDG pore that is intrinsic to the P2X7 channel are two studies by Yan et al. who carefully investigated the complex biphasic current responses observed upon prolonged activation of P2X7 receptor which consist of a fast current increase in the millisecond range and a slowly increasing high-amplitude current that peaked after tens of seconds and had been associated with the presence of two ATP binding sites of different affinity [126]. Yan et al. showed that this slow current component temporally coincided with the shift in reversal potential in NMDG-containing extracellular solutions, and an immediate NMDG permeability was observed when residue Thr 15 in the P2X7 N terminus was mutated to residues with larger side chains (Glu, Lys, or Trp) [257]. Based on the activation and deactivation kinetics at different agonist concentrations and on the sensitization properties of the P2X7 receptor, a gating model was proposed in which occupancy of ATP binding sites controls channel conductance [258]. Involvement of the N-terminus in generation of an NMDG-permeable pore is further suggested by the properties of a P2X7 N-terminal splice variant, which also shows immediate NMDG permeability [300]. In conclusion, although the processes of pore dilation are still very poorly understood, the above data suggest that NMDG permeability increases in P2X2, P2X4, and P2X7 receptors are based on a common molecular mechanism intrinsic to the ion channels. Whether this dilated I_2_ state is also responsible for dye uptake remains a matter of debate [301].

Inconsistent with an intrinsic channel property, however, is the fact that neither for P2X2 and P2X4 receptors nor for P2X7 receptors permeability states corresponding to the dilated channels have been observed in single channel recordings [172, 287, 302], and in P2X2 receptors, pore formation properties varied between experiments [287]. This could in part be due to the fact that optimal conditions for production of the I_2_ state (very low extracellular Ca^2+^ and concentrations of ATP >10 μM) [283, 285] might not have been systematically explored.

Interestingly, a G496A polymorphism in the human P2X7 receptor [303] has been identified that produced loss of function in flow cytometry assays (ethidium uptake, apoptosis, Ba^2+^ influx) but revealed no differences in the electrophysiological properties of the heterologously expressed channel [304]. Likewise, conflicting data were found for the L451P polymorphism in the mouse P2X7 receptor which showed reduced pore formation and cell death if native thymocyte or T cell preparations were investigated by flow cytometry [305, 306], but no difference to wild-type (wt) when heterologusly expressed variants were analyzed in fluorometry imaging assays [79] or in patch clamp experiments (own unpublished data). These data support the involvement of cell-specific and C terminus-dependent mechanisms in the formation of dye-permeable pores.

### P2X7-specific signalling

A range of downstream cellular events have been identified upon P2X7 receptor activation. These include release of cytokines, cytoskeletal rearrangements, and plasma membrane protein shedding and cell death via necrosis and apoptosis as well as trophic effects, cell proliferation, and differentiation (e.g., [293, 307–309]). The cellular mechanisms underlying these various effects are dependent on the cell background and are very incompletely understood. Interactions with multiple intracellular signalling pathways have been shown. These include activation of phospholipases A2 and D and coupling to protein kinases PKC, Src, JNK (stress-activated protein kinase) and the ERK and p38MAP kinases, as well as the rho-associated protein kinase (ROCK). For a comprehensive picture of P2X7 signalling in microglia, refer to Kettenmann et al. [310]. For additional information, refer to Erb et al., Duan et al., and Lenertz et al. [311–313]. Only two of the above-mentioned effects are briefly mentioned in the following.

#### Secretion of IL-1β and other cytokines

The cytokine IL-1β is released by macrophages and other immune cells and represents an important mediator of inflammation. It is transcribed as the inactive precursor pro-IL-1β in response to inflammatory stimuli (e.g., LPS that acts via TLR and NFkB activation). Processing of pro-IL-1β into IL-1β involves the protease caspase-1, which in turn is proteolytically generated from procaspase-1 in a process that involves different multimeric "inflammasome complexes" that oligomerize and activate caspase-1 in response to a second specific stimulus [314, 315]. These can be the toxins nigericin and maitotoxin or high ATP concentrations in case of the NALP3 inflammasome [316]. Early studies had already shown that ATP is an efficient stimulator of IL-1β maturation and release [317–320] and that K^+^ efflux appears to be involved in this process [320]. By generation of the P2X7−/− mouse [321–323], it was confirmed that the IL-1β release (and release of other IL-1 family members) is a consequence of P2X7 receptor activation. For a detailed review, see Ferrari et al. [324].

However, the molecular mechanisms that lead from K^+^ depletion to NALP3 inflammasome activation and IL-1β release and how pannexin is involved in this process remain unclear [301, 325]. P2X7-mediated microvesicle shedding [326, 327] has been suggested to play a role in interleukin release.

#### Effects on extracellular and intracellular membranes

A variety of changes in the plasma membrane composition and morphology have been observed upon P2X7 receptor activation [327]. These include the redistribution of phosphatidylserine to the extracellular leaflet of the plasma membrane (PS-flip), plasma membrane protein shedding (CD26L, CD23, CD 27) by matrix metallo proteases, and plasma membrane blebbing and microvesicle release. The so-called PS-flip represents an indicator of apoptotic cell death but has also been involved in physiological processes such as maturation and differentiation. In addition, P2X7 receptors have effects on intracellular organelles and membranes. For example, regulation of phagosome fusion with lysosomes has been shown to be involved in bacterial killing (for details on P2X7 regulation of extracellular and intracellular membrane responses refer to Qu and Dubiak [327]).

## Physiological functions of P2X receptors inferred from genetically modified animals and other in vivo models

P2X receptor isoforms have been found to be widely but specifically distributed among different tissues in the vertebrate body. Expression patterns of P2X receptors have initially been evaluated at the mRNA level using Northern blot, RT-PCR, and in situ hybridization analysis [23, 61, 205, 328–330]. Following the development of P2X receptor subtype-specific antibodies, many of these findings have been verified at the protein level by Western blotting and immunohistochemistry [331–337]. It has to be mentioned, however, that reliability of some of these antibodies have been questioned [338, 339]. In addition, the lack of subtype-selective and metabolically stable agonists as well as truly potent and specific antagonists for some P2X subtypes has made the molecular identification of individual P2X receptor subtypes in native tissue preparations and determination of their function a challenging task. Thus, the identities and in vivo roles of many P2X receptors are still not completely understood or speculative. Dramatic progress has been made by the use of knockout and transgenic animals. The following part of this review will provide an overview of the P2X receptor distribution and their physiological roles determined using both genetically engineered animal models and transient knockdown approaches (Table 1).

**Table 1:** Major physiological functions of different P2X receptor subtypes determined using knockout (KO), P2X2/P2X3 double knockout (DKO), and transgenic mice (TG) mice

Subtype	Phenotype (mouse model)	Physiological function	Reference
P2X1	Male infertility (KO)	Smooth muscle contraction	[344]
	Impaired kidney function (KO)	Renal autoregulation	[355–357]
	Reduced arterial thrombosis (KO)/prothrombotic phenotype (TG)	Platelet activation	[361, 362]
P2X2	Impaired synaptic facilitation (KO)	Regulation of transmitter release in hippocampus	[275]
	Reduced inflammatory pain (KO, DKO)	Nociceptive signalling	[65]
	Impaired peristalsis in small intestine (KO)	Intestinal neurotransmission	[394]
	Attenuated ventilatory response to hypoxia (KO, DKO)	Carotid body function	[396]
	Urinary bladder hyporeflexia (KO, DKO)	Sensory neurotransmission	[65]
	Impaired taste sensing (DKO)	Gustatory signalling	[433–435]
	Abnormal skeletal neuromuscular junctions (KO)	Endplate formation	[399]
P2X3	Impaired hippocampal LTD (KO)	Regulation of synaptic plasticity	[426]
	Reduced pain responses (KO, DKO)	Nociceptive signalling	[65, 416, 417]
	Impaired peristalsis in small intestine (KO)	Intestinal neurotransmission	[423]
	Urinary bladder hyporeflexia (KO, DKO)	Sensory neurotransmission	[65, 416]
	Impaired temperature sensitivity (KO)	Thermal sensation	[417, 425]
	Impaired taste sensing (DKO)	Gustatory signalling	[433–435]
P2X4	Decreased hippocampal LTP (KO)	Regulation of synaptic plasticity	[455]
	Reduced inflammatory and neuropathic pain (KO)	Modulation of chronic pain (regulation of BDNF and PGE2 release from activated microglia/macrophages)	[191, 460, 461]
	Higher blood pressure, lack of vascular remodelling, decreased flow-induced release of NO (KO)	Regulation of vascular tone	[456]
	Improved heart function (TG)	Control of contractility of the cardiomyocytes	[468–472, 474]
P2X7	Abolished IL-1β release, diminished inflammatory responses (KO)	Pro-inflammatory cytokine release	[322, 323]
	Reduced inflammatory and neuropathic pain (KO)	Immune cells activation	[321]
	Skeletal abnormalities (KO)	Bone metabolism	[552, 553]
	Reduced fluid secretion in salivary gland and pancreas (KO)	Regulation of exocrine gland secretion	[556, 557]

### P2X1

The P2X1 receptor sequence was originally cloned from a rat vas deferens cDNA library [23] and subsequently isolated from human urinary bladder and platelets as well as mouse urinary bladder and vas deferens [340–343]. This receptor is most highly expressed in smooth muscle cells of various organs, including urinary bladder, vas deferens, and arteries [331, 336, 344–346]. Significant P2X1 receptor levels are also found in megakaryocytes and blood platelets [342, 347, 348]. P2X1 mRNA has been detected in apoptotic thymocytes but, surprisingly, not in peripheral T cells [349]. The precise localization of P2X1 receptors in the CNS remains unsettled as the P2X1 receptor antibodies were shown to exhibit similar immunostaining patterns in the CNS of wt and P2X1 knockout mice [338]. However, contribution of P2X1 subunits to functional responses in cortical astrocytes was reported [67, 350], and P2X1 receptor expression, detected by immunostaining, was shown to be downregulated in the hippocampus by TNP-ATP treatment [351]. Peritoneal mouse macrophages were shown to express functional P2X1 receptors absent in P2X1−/− mice [352]. In addition, human lung mast cells were shown to express functional P2X1 receptors [330].

**Fig. 4:**
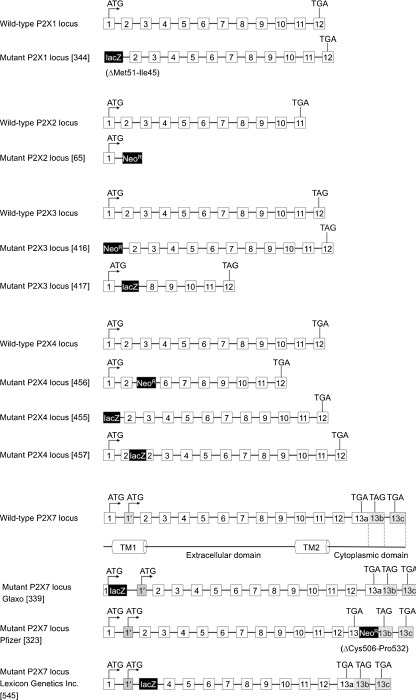
Summary of published P2X receptor−/− mice and the targeting strategies used for their generation. Location of TM encoding exons are only shown for the P2X7 receptor, but, due to the strong conservation of exon-intron boundaries in P2X receptor encoding genes, can be transferred to the other subtypes. The figure also illustrates alternative exons (1´and 13b/c) identified in the rodent P2X7 gene [300, 525] and explains how the P2X7K splice variant derived from exon 1´ can escape the gene deletion strategy used in one of the available P2X7−/− mouse lines. Note that all cassettes indicated with lacZ represent actually lacZ-Neo^R^ cassettes

The P2X1 encoding gene consists of 12 exons and is located close to the P2X5 gene on chromosome 10 in the rat, chromosome 17 in the human, and chromosome 11 in the mouse genome [340, 341, 343]. In most cases, the distribution profiles described for P2X1 receptors correlate with phenotypic data from P2X1 loss- and gain-of-function mouse models. P2X1-deficient mice were generated through the targeted replacement of a fragment encoding a part of exon 1 (including the initiation codon ATG) by a lacZ/Neo cassette [344] (Fig. 4). In line with an important function in the vas deferens, these knockout mice revealed a drastic (up to 90%) reduction in male fertility. This was associated with a low sperm count in the ejaculate caused by the lack of P2X1 receptor-mediated vas deferens contraction [344]. Consistent data have been reported in guinea pig, rat, and human by functional and pharmacological analysis of isolated vas deferens preparations [6, 353, 354].

Renal P2X1 receptor-mediated pressure-induced afferent arteriolar autoregulatory responses were studied in P2X1−/− mice using the in vitro blood perfused juxtamedullary nephron technique [355–357]. It was suggested that P2X1 receptors expressed on afferent arteriolar smooth muscle cells contribute to the modulation of the tubuloglomerular feedback mechanism (TGF). Accordingly, macula densa cells of the juxtaglomerular apparatus respond to the increase in luminal tubular fluid NaCl concentration through release of ATP that, via the P2X1-mediated vasoconstriction of afferent arterioles, leads to changes in the glomerular filtration rate [331, 355–358]. These in vitro findings were confirmed in vivo by data from whole kidney blood flow experiments that show pharmacologically the importance of the P2X1 receptor activation for whole kidney autoregulation [359]. However, a recent study indicates that the direct activation of P2 purinergic receptors by ATP is not a major cause of TGF-induced vasoconstriction in vivo [360]. Therefore, additional studies are required to fully understand the involvement of P2X1 receptors in renal hemodynamics.

Analysis of the P2X1 knockout mice further indicated an involvement of P2X1 receptors in platelet function and thrombus formation [361]. Mice lacking the P2X1 receptor display reduced arterial thrombosis under conditions of high shear stress and exhibit reduced platelet aggregation in low collagen concentrations. About 20% of the analyzed P2X1−/− mice showed also markedly prolonged bleeding times [361]. Involvement of the P2X1 receptor in platelet function was also shown in a P2X1 transgenic mouse model in which human P2X1 cDNA was overexpressed under the control of the megakaryocyte-specific murine glycoprotein IIb (GPIIb) promoter [362]. This resulted in a mild prothrombotic phenotype. Accordingly, transgenic platelets displayed enhanced secretion and aggregation in vitro, in response to low doses of collagen, convulxin, and the thromboxane A2 mimetic U46619 or shear stress [362]. These observations are consistent with in vivo results obtained in a model of pulmonary thromboembolism, which demonstrated increased lethal thrombosis in hP2X1 transgenic mice compared with wt animals [362]. The involvement of P2X1 receptors in platelet activation during hemostasis or thrombosis make it an important therapeutic target. For more detailed information concerning P2X1 receptor function in platelet, see Hu et al. and Mahaut-Smith et al. [363, 364].

### P2X2

The P2X2 cDNA was initially cloned from a rat pheochromocytoma PC12 cell line [22]. The P2X2 receptor is one of, if not the, most widely distributed subtype of the P2X receptor family. Its abundant expression has been found in both the central and the peripheral nervous systems [278, 365]. Particularly high expression levels were described in the olfactory bulb, cerebral cortex, basal ganglia, diencephalon, mesencephalon, cerebellum, medulla oblongata, and dorsal horn area of the spinal cord [200, 335, 337, 366–369]. Moreover, a significant P2X2 expression with importance for sensory neurotransmission has been described in both sensory and autonomic ganglion neurons of the peripheral nervous system [65, 200, 368, 370–378]. In addition, multiple non-neuronal tissues, such as adrenal medulla (chromaffin cells), urothelium, vasculature smooth muscle, skeletal muscle (during development and regeneration), cardiac muscle, and interstitial cells of the vas deferens, have been shown to express significant amounts of the P2X2 receptor subunit [22, 336, 337, 345, 379–385].

The P2X2 gene comprises 11 exons and lies together with the P2X4 and P2X7 genes on chromosome 12 in the rat and human and on chromosome 5 in the mouse genome. Multiple alternatively spliced transcripts of the P2X2 receptor have been detected [115, 368, 386–393]. The described variants arise from C-terminal alternative-splicing events. The functional, but fast desensitizing P2X2b (or P2X2-2) receptor isoform, in which exon 11 is partially deleted, appears to be conserved between human and rodents. Other identified splice variants include P2X2c, P2X2d, P2X2e, P2X2f, and P2X2g in rat [115, 368, 386, 387, 389], P2X2e in mouse [391, 392], P2X2-3 in guinea pig [388, 390], and P2X2c and P2X2d in human [393]. Some of these appear to be non-functional. It seems likely that various P2X2 receptor isoforms co-assemble with other P2X subunits to form heteromeric channels with modified properties [391].

Mice lacking the P2X2 subunit were generated by targeted deletion of a region spanning from exon 2 to exon 11 and its replacement with a floxed sequence encoding a neomycin resistance gene [65, 275] (Fig. 4). Despite the wide distribution of the P2X2 receptor, P2X2 knockout mice displayed only small differences in body weight compared with wt animals and were visibly and histopathologically normal for up to 1 year of age. Likewise, urinalysis, blood chemistries, and peripheral blood cell counts did not show significant differences between P2X2+/+ and P2X2−/− mice [65].

Analysis of P2X2 knockout mice revealed impaired peristalsis in the small intestine, most likely due to the absence of P2X2 receptor-mediated synaptic transmission in the myenteric plexus [394] but not in the mouse colon [395], indicating that the P2X2 subunit is not required for propulsive motility in the mouse colon. Also, an involvement in ATP-evoked synaptic facilitation of hippocampal interneurons [275] was reported, and a pivotal role of P2X2 subunit containing receptors in normal carotid body function and in ventilatory response to hypoxia was demonstrated in P2X2−/− mice. The mice showed reduced responses of the carotid sinus nerve to hypoxia and markedly attenuated ventilatory responses to hypoxia [396].

An involvement of P2X2-subunit-containing receptors in sensory transmission has early been proposed [397] and was confirmed in P2X2−/− mice. In agreement with the co-expression of P2X2 and P2X3 subunits on DRG neurons, functional analysis of wt and P2X2−/− mice revealed the contribution of P2X2 subunits to ATP-induced sustained but not transient (fast desensitizing) responses of DRG and nodose ganglia neurons. Sympathetic neurons of the superior cervical ganglion from P2X2−/− mice exhibited no response to αβ-me-ATP, indicating an exclusive expression of P2X2 subunits in the form of homomeric P2X2 receptors [65], which is in good agreement with former pharmacological studies of cultured autonomic ganglia neurons [376, 398]. In the formalin-induced model of chemical nociception, P2X2 subunit deficiency leads to significant attenuation in the persistent but not acute phase of the formalin response [65]. Also, urinary bladder hyporeflexia and decreased activities of pelvic afferent nerves in response to bladder distension were observed in these mice [65].

Further experiments with P2X2 knockout mice have demonstrated a role for P2X2 receptor-dependent signalling in the development and maintenance of skeletal neuromuscular junctions [399]. The involvement of P2X2 receptor in the late stages of endplate formation is consistent with the expression pattern of the P2X2 subunit during skeletal muscle development and muscle fiber regeneration [382–384].

### P2X3

The P2X3 sequence was originally cloned from a rat DRG cDNA library [400, 401] and subsequently also from a human heart cDNA library [88] and mouse genomic library [402]. In rodents, predominant and developmentally regulated P2X3 receptor expression has been demonstrated on small- to medium-diameter sensory neurons within DRG as well as nodose and trigeminal ganglia by Northern-blot analysis, in situ hybridization or immunohistochemistry [373, 374, 380, 400, 401, 403–408]. This very restricted pattern of P2X3 subunit distribution has been associated with P2X3-mediated nociceptive sensory nerve responses to ATP released from inflamed or damaged tissues [187, 333, 409]. The P2X3 subunit has also been detected in the spinal cord within the superficial laminae of the dorsal horn [373, 385, 404, 405]. In addition, P2X3 receptor expression has been found in both the urothelium and suburothelium of rat urinary bladder [410]. In humans, the P2X3 receptor has so far been reported in heart and spinal cord at the mRNA level and in dorsal root ganglia, intestine (myenteric plexus neurons), urinary bladder (urothelium and suburothelium), and dental pulp at the protein level [88, 410–415].

The gene encoding the P2X3 subunit contains 12 (human and mouse) to 13 (rat and zebrafish) exons and was mapped to chromosome 3 in rat, chromosome 2 in mouse, and chromosome 11 in human [88, 402].

An important role of the P2X3 channel in nociceptive signalling was confirmed using two independently engineered P2X3 knockout mouse lines. One line was generated by targeted replacement of the fragment, containing the ATG translational start site and exon 1, with a floxed neomycin-resistance gene [416]. In a second strain, a fragment ranging from exon 2 to exon 7 was replaced by an IRES-LacZ-MC1-Neo cassette [417] (Fig. 4). Both mice strains display significantly attenuated responses in the acute and persistent phases of the formalin-induced pain test [416, 417]. Likewise, pain caused by intraplantar injection of ATP is also greatly diminished in the P2X3−/− mice [416]. A significant role of P2X3 receptors in pain responses with reduction of agonist-induced mechanical hyperalgesia and tactile allodynia as well as reduced pain responses in neuropathic or inflammatory pain models is in agreement with studies using P2X3 siRNA [418, 419], antisense oligonucleotides [420], or pharmacological P2X3 receptor inhibition [141] in rats.

Deletion of the P2X3 gene in mice also had a significant effect on sensory function in the urinary bladder as evidenced by marked bladder hyporeflexia, resulting in greatly reduced voiding frequency and substantial increase in bladder capacity [65, 416]. Further studies demonstrated attenuated responses of pelvic afferents to bladder distension and intravesical injection of P2X agonists (ATP or α,β-meATP) in P2X3−/− mice [421]. It was concluded that bladder filling and subsequent distension induces release of ATP from the urothelium, which, via P2X3 receptors, triggers the mechanosensory signal transduction and excitation of afferent nerve fibers [421, 422]. Similar to the P2X2 null mutation, P2X3 deficiency also resulted in impaired peristalsis in the small intestine [423] but not in the mouse colon [395]. In addition, P2X3−/− mice exhibited a blunted response of gastric vagal afferents to fluid distension of oesophagus and stomach [424].

In line with an important function in sensory systems, both P2X3−/− models also showed an enhanced thermosensory phenotype [417, 425] and were unable to differentiate the intensity of non-noxious ‘warming’ stimuli [417]. However, it should be noted that thermal hyperresponsiveness observed in P2X3 null mice could not be reproduced by subcutaneous administration of A317491, a P2X3-selective antagonist [425]. Therefore, it has been suggested that long-term absence of P2X3 receptor is necessary to develop such a thermosensory phenotype or that compensatory changes contribute, at least in part, for the P2X3−/− phenotype [425]. Finally, analysis of synaptic plasticity in P2X3−/− mice has indicated that P2X3 receptor might be involved in the induction of long-term depression (LTD) at hippocampal synapses [426].

Besides the mouse models mentioned before, analyses of P2X3 receptor function has been performed in the zebrafish model system. Three groups reported the identification of orthologues of the mammalian P2X3 subunit in zebrafish [120, 427, 428]. The zebrafish orthologue, zP2rx3a (zP2X3, also termed zP2rx3.1), is located on chromosome 14, whereas zP2rx3b (or zP2rx3.2) is located on chromosome 1 [429]. Similarly to mammals, the expression of the zP2X3 receptor has been mainly detected on sensory neurons. Very high levels were found in neurons of the trigeminal ganglia but also in the Rohon-Beard cells of the dorsal spinal cord [428, 429]. A non-neuronal expression of zP2X3 receptor has been found within lateral cranial ectodermal cells in zebrafish embryos [430, 431]. In order to characterize the function of zP2X3 receptor during development, morpholino oligonucleotide-mediated knockdown of this channel was performed [430]. These studies revealed that the zP2X3 subunit is required for normal craniofacial development and sensory neurogenesis because its loss in embryos led to craniofacial defects, such as malformation of pharyngeal skeleton and disrupted epibranchial ganglia formation. The specificity of this phenotype was confirmed by additional morpholino oligonucleotides, which target another sequence of the zP2RX3 gene and by partial rescue of the mutant phenotype by co-injection of rP2X3 RNA [430].

P2X2 and P2X3 subunits are co-localized in many neurons, particularly within dorsal root ganglia, nodose ganglia, nucleus tractus solitarius, and taste buds [373, 380]. Double knockout P2X2/P2X3^Dbl−/−^ mice were generated by breeding the compound heterozygous offsprings obtained upon crossing of P2X3−/− (lacking the ATG translational start site and exon 1 [416]) with P2X2+/− mice [65]. Surprisingly, about 90% of these P2X2/P2X3^Dbl−/−^ mice died in the early postnatal period with various abnormalities including: distended bladders, enlarged hearts, pronounced atrophy or hypocellularity of lymphohematopoietic organs, and lack of lymphoid follicles in the spleen and mesenteric lymph node [65]. Bacterial bronchial pneumonia was identified as the leading cause of mortality. Those P2X2/P2X3^Dbl−/−^ mice that survived into adulthood (approximately 10%) were found to be normal in appearance and weight. In contrast to P2X2−/−, but similarly to P2X3−/− mice, the survived double mutant mice had reduced pain behavior in both phases of the formalin test [65] and provided evidence that the P2X2 and P2X3 subunits are the predominant P2X family members on mouse sensory neurons, since their deletion led to loss of virtually all ATP-activated currents in DRG and nodose ganglia.

Consistent with findings in P2X2 and P2X3 single knockout mice, P2X2/P2X3^Dbl−/−^ mice developed urinary bladder hyporeflexia, decreased pelvic afferent fiber activity in response to bladder filling [65], and reduction in pelvic afferent response to colorectal distension [432]. Further studies highlighted a crucial role of the P2X2 subunit in ventilatory response to hypoxia, since this is significantly decreased in both P2X2−/− and P2X2/P2X3^Dbl−/−^ mice but not in mice deficient in only the P2X3 receptor [396].

Finally, studies on P2X2/P2X3 ^Dbl−/−^ mice showed that ATP serves as a primary neurotransmitter in taste buds [433]. P2X2/P2X3^Dbl−/−^ mice displayed a loss of peripheral gustatory nerve responses to salt, sweet, sour, bitter, and umami [433, 434]. Despite this profound taste deficit, avoidance of caffeine, and citric acid was comparable to that observed in wild-type controls, suggesting involvement of either non-gustatory or non-purinergic mechanisms for these taste stimuli [433, 435]. In contrast to double mutant mice, single P2X2−/− or P2X3−/− animals exhibited only moderate changes in taste-mediated behavior, suggesting that homomeric P2X2 or homomeric P2X3 receptors suffice for normal taste function [433].

Collectively, all these findings demonstrate that homomeric P2X2, homomeric P2X3, and heteromeric P2X2/3 receptors are crucial players in sensory neurotransmission.

### P2X4

The first P2X4 receptor cDNAs were cloned from hippocampus, whole brain, and superior cervical ganglia cDNA libraries [87, 436, 437]. Additional P2X4 sequences were cloned from various species and tissues [328, 438–443] including *X*. *laevis* oocytes and zebrafish [57, 444]. A characteristic feature of the P2X4 subunit is its widespread distribution that overlaps to a large extent with the localization pattern of the P2X6 subunit [200]. An extensive and abundant P2X4 receptor expression has been demonstrated in several regions of the central and peripheral nervous systems [87, 335, 369, 374, 436, 437, 439, 445] as well as all vital and reproductive organs, skeletal and smooth muscle, epithelial and endothelial cells, and various others [87, 381, 436–438, 441, 442, 445–452]. The gene encoding the human P2X4 receptor comprises 12 exons and is located about 24 kilobases downstream of the P2X7 receptor gene. This close chromosomal localization suggests that P2X4 and P2X7 genes evolved by gene duplication. Multiple splice variants, often with distinct patterns of expression, have been found for the P2X4 receptor [441, 443, 453]. A putative minimal promoter of the human P2X4 gene was identified and shown to be regulated by the hematopoietic transcription factor GATA-2 [454].

The physiological role of P2X4 receptors has been assessed using a variety of approaches, including knockdown with either antisense oligonucleotides or small interference RNA as well as three P2X4−/− mouse lines (Fig. 4). In the first knockout strategy, a lacZ/Neo cassette was used to replace a region encompassing the first P2X4 exon (including the ATG start codon) and a short part of the first intron [455]. In a second KO model, the P2X4 genomic fragment containing exons 3, 4, and 5 was replaced with a loxP-flanked neomycin resistance cassette [456]. In the third P2X4 knockout mouse line, a short fragment within exon 2 was replaced by an IRES-lacZ/Neo cassette [457].

Numerous studies have shown the involvement of the P2X4 receptor in the pathogenesis of chronic neuropathic and inflammatory pain. P2X4 expression is increased in microglia of the dorsal horn following spinal nerve ligation, a model of neuropathic pain [190], and intrathecal administration of P2X4 antisense oligodeoxynucleotides into rats significantly attenuated both spinal cord-induced tactile hypersensitivity as well as the increase in P2X4 receptor levels [190]. Likewise, tactile allodynia was reversed by spinal administration of TNP-ATP [190]. The increase of P2X4 receptor expression was essentially restricted to hyperactive microglia within the spinal dorsal horn and injection of ATP-stimulated microglia into normal rats resulted in P2X4-dependent tactile allodynia. In agreement with these data, spinal cord injury or formalin injection into the hindpaw of rats resulted in an increase in P2X4 receptor expression on microglial cells [458, 459]. P2X4−/− mice lines [455, 460, 461] showed a marked decrease in tactile allodynia caused by nerve injury and a significant reduction in peripheral inflammation-induced pain while behavioral responses to acute thermal, mechanical, and chemically induced pain appeared normal. It was demonstrated in two P2X4−/− lines that P2X4 receptors control the release of BDNF from activated microglia, which is promoting allodynia [191, 460]. Further analysis demonstrated that the reduction of inflammatory pain behavior in P2X4-deficient mice was due to impaired synthesis of prostaglandin E2 (PGE2), a central mediator of inflammation involved in pain hypersensitivity [461]. Accordingly, wild-type, but not P2X4−/−macrophages, exhibited elevated levels of PGE2 after ATP-mediated activation of the P2X4 receptor. For further information, see Jakobsson [462].

In both KO lines [460, 461], no differences to wt animals were observed in the formalin test of injury-induced pain, when standard amounts of formalin were used. However, injection of a lower amount of formalin into a mouse paw revealed that the second phase of the formalin test, which is attributed to chronic inflammatory response, was markedly attenuated in P2X4-deficient mice [461]. In contrast, the first phase of the formalin test, representing acute nociception in response to primary afferent activity, was unaffected in P2X4 null mice. This finding indicates that P2X4 channels are not involved in acute nociception but most likely play a role in chronic inflammatory pain that is determined by both peripheral and central sensitization. Also, a third P2X4−/− model [457] revealed the presence of functionally active P2X4 receptors in native peritoneal macrophages. Downregulation of the P2X4 receptor by knockdown approaches [463, 464] as well as its deletion [456] have also resulted in abnormal endothelial cell responses to changes in blood flow, including a lack of flow-induced Ca^2+^ influx and diminished production of the endogenous nitrovasodilator, nitric oxide (NO). Accordingly, P2X4−/− animals exhibited an elevated blood pressure, a lack of vascular remodelling, and decreased flow-induced release of NO, suggesting a crucial role of P2X4 channel in endothelial cell-mediated control of the vascular tone. Involvement of the P2X4 receptor in control of Ca^2+^ entry in vascular endothelial cells exposed to shear stress was shown [465]. A P2X4- or P2X6- mediated Ca^2+^ influx in response to extracellular Zn^2+^ and ATP has later been shown to restore Cl^−^ secretion across cystic fibrosis airway epithelia, suggesting P2X4 and/or P2X6 receptors as a potential therapeutic targets for the treatment of cystic fibrosis [466, 467].

P2X4 receptors have been immunohistochemically identified in perisynaptic locations on hippocampal CA1 and cerebellar Purkinje cells [335], and analysis of P2X4−/− mice revealed a significant decrease in LTP at Schaffer collateral synapses. Moreover, ivermectin had no effect on P2X4−/− animals, whereas it enhanced LTP in wild-type controls, thus indicating involvement of P2X4 receptor-mediated Ca^2+^ influx in regulation of hippocampal synaptic plasticity [455].

The role of the P2X4 receptor in the heart was studied in a transgenic mouse model overexpressing the human P2X4 subunit under the control of the cardiac-specific α-myosin heavy chain promoter [468, 469]. Although no apparent histopathological abnormalities were observed under normal physiological conditions, the hP2X4 transgenic mice exhibited increased contractility of the cardiomyocytes and greater global contraction performance in intact heart as compared with wt mice [468]. Therefore, it was hypothesized that P2X4 receptor activation may be beneficial in pathophysiological conditions, such as cardiomyopathy and ischemic heart disease. In order to test this possibility, binary P2X4 receptor (P2X4)/calsequestrin (CSQ) transgenic mice were generated by crossing hP2X4 transgenic mice with a CSQ transgenic mouse model of cardiomyopathy [470]. Interestingly, overexpression of the P2X4 receptor in the P2X4/CSQ mutant mice resulted in significant delay of heart failure progression and a more than twofold increase in life expectancy [470–472]. Similarly, chronic in vivo administration of the P2 receptor agonist MRS-2339 was found to not only reduce cardiac hypertrophy and prolong survival of the CSQ single transgenic mice but also to improve cardiac function in dogs with tachycardia-induced cardiomyopathy, thus emphasizing an important role of cardiac P2X channels in heart physiology [471, 473]. Further evidence for a rescue effect of overexpressed hP2X4 receptors in ischemic heart failure was provided in an analysis of hP2X4 receptor overexpressing transgenic mice after myocardial infarction [474], suggesting that the P2X4 channel represents a therapeutic target for the treatment of heart failure resulting from ischemia.

In zebrafish, two P2X4 genes, designated zP2rx4a (zP2rx4.1, chromosome 21) and zP2rx4b (zP2rx4.2, chromosome 8), have been identified, and the zP2rx4b transcript has been localized in the embryonic nervous system, including dorsal and ventral neurons of spinal cord and the trigeminal nerve [429]. While no information is available concerning the expression pattern of the zP2rx4a receptor, its successful crystallization has greatly furthered our understanding of the molecular structure of the P2X channel family [48].

### P2X5

A cDNA clone encoding the P2X5 receptor was originally isolated from rat celiac cervical ganglia and shortly afterwards also from a rat heart cDNA library [200, 201]. Corresponding sequences were later obtained from human brain, chicken embryo skeletal muscle (initially named cP2X8), brain and heart cDNA libraries, a mouse BAC library, bullfrog tadpole skin, and zebrafish [56, 57, 59–61, 124, 202, 429, 475].

The gene encoding the P2X5 subunit consist of 12 (chicken, human) to 13 (mouse, rat) exons and shares its chromosomal localization with the P2X1 gene (see section “P2X1”) [56].

The murine P2X5 channel appears to be widely distributed in the central and enteric nervous systems [476, 477]. In addition, it has been detected in cardiac and skeletal muscle, adrenal gland, kidney, and testis [56, 61, 201, 478]. In humans, P2X5 receptor expression has been found to be predominant in the immune and central nervous systems [60]. In contrast to other vertebrate species, two alternative splice variants of the P2X5 receptor have been reported in humans, P2X5a, that lacks exon 10, and P2X5b, missing exons 3 and 10 [19, 60]. The P2X5a transcript results from a single nucleotide polymorphism (SNP) at the 3’ splice site of exon 10 and thus encodes a truncated, non-functional P2X5 protein lacking a portion of both the putative ATP binding site and TM2 [55, 479]. The allele encoding this non-functional P2X5 isoform is the most prevalent variant in different human populations [479].

Interestingly, several reports have linked P2X5 receptor expression with differentiation and turnover of various cell types, such as skeletal muscle cells, osteoblasts, and epithelial cells from different tissues (nasal mucosa, skin, vagina, gut, bladder, ureter, thymus) [381, 383, 447, 480–485]. Ryten et al. provided evidence that ATP-mediated activation of P2X5 receptors suppresses proliferation and promotes differentiation of skeletal muscle progenitor cells (known as satellite cells) into muscle fibers [485]. Therefore, the P2X5 channel has been postulated to play a role in skeletal muscle development or regeneration [485, 486]. Consistent with this, developmentally regulated expression of the P2X5 receptor has been reported in rat and chick skeletal muscle [383, 484]. In addition, relatively high P2X5 receptor expression has been found in different cancer tissues, including basal and squamous cell carcinomas as well as prostate cancers, indicating that activation of P2X5 receptor may also regulate growth and differentiation of cancer cells [487–489].

P2X5 knockout or transgenic animals have not been described so far. However, data from siRNA-mediated knockdown of P2X5 receptors in human bronchial epithelial cells and the fact that most humans express the non-functional P2X5 isoform indicate that at least, in humans, it does not fulfil an essential physiological function [465, 479].

In zebrafish, two paralogous P2X5 genes have been reported. zP2rx5.1 was mapped on chromosome 5, and zP2rx5.2 (also known as zP2rx514 or zP2rx8) is located on chromosome 15 [57, 429, 490]. The zP2rx5.1 receptor is the only zP2X receptor for which mRNA has been detected in embryonic skeletal muscle, whereas zP2rx5.2 mRNA, like other zP2X receptor mRNAs, was found in the nervous system [429, 490, 491]. Using morpholino-mediated zP2rx5.1 gene knockdown, it was demonstrated that the zP2rx5.1 receptor is necessary for the muscle responsiveness to ATP but is not essential for myogenesis during zebrafish embryonic development [490].

### P2X6

The P2X6 receptor sequence was first cloned from the rat superior cervical ganglia and shortly afterwards from a rat brain cDNA library [200, 205]. Subsequently, human and murine P2X6 receptor counterparts (originally designated as P2XM) have been identified in a search for novel p53-regulated genes [206, 492]. Interestingly, no P2X6 ortholog has so far been found in zebrafish or any other non-mammalian species [490].

Northern blot analyses have demonstrated a predominant expression of murine and human P2X6 transcripts in skeletal muscle [206, 492]. Further studies have shown that expression of the P2X6 receptor in chick, rat, and mouse skeletal muscle, similarly to the P2X5 receptor, is regulated during embryonic development [382, 383, 484]. In addition, widespread distribution of rat P2X6 receptors, overlapping to a large extent with the expression pattern of P2X4 and P2X2 subunits, has been reported in both the central and the peripheral nervous systems [200, 335, 493, 494]. Furthermore, the P2X6 subunit has been found, often together with the P2X4 subunit, in epithelial cells of various organs (renal tubule, bronchi, thymus, umbilical vein) but also in gland cells of the uterus and granulosa cells of the ovary [200, 270, 336, 381]. It is well established that the P2X6 subunit is unable to homo-oligomerize effectively, and the frequent co-localization of P2X6 with P2X4 or P2X2 subunits suggests the formation of heteromeric P2X2/6 and P2X4/6 channels [45, 203, 204, 495].

The P2X6 gene comprises 12 exons and lies on chromosome 16 in mouse, chromosome 11 in rat, and chromosome 22 in humans [492]. Four alternatively spliced transcripts, partially showing different expression patterns, have been described for the human P2X6 receptor [465, 492, 496]. In the first splice form, designated AL1, a portion of exon 1, encoding a part of the TM1, is eliminated. The second splice variant, AL2, lacks exon 10, whereas the third splice form, AL3, misses exons 10–11, resulting in truncated P2X6 proteins lacking the TM2 [492, 496]. A fourth splice variant of the P2X6 receptor, lacking exon 4, has later been found together with the full-length transcript in human CF and non-CF airway epithelial cell lines [465]. Interestingly, aberrant splicing patterns or even no P2X6 mRNA expression have been demonstrated in various soft tissues sarcomas, suggesting a role for P2X6 receptor in tumorigenesis [496].

An alternatively spliced P2X6 variant has also been identified in mouse. This splicing product misses exon 8 and most probably gives rise to a non-functional protein, which lacks a portion of the extracellular loop, the entire TM2, and the intracellular domain [382, 497]. Although both the full-length P2X6 transcript and the alternatively spliced form are present during mouse postnatal development and in adult brain, the expression level of the full-length form is much higher. In contrast, the splice variant appears to be the predominant form expressed during neuronal differentiation of P19 embryonal carcinoma cells [497]. The functional significance of this alternative splice product in mice is not clear, but it might regulate P2X6 receptor activity during the process of neuronal differentiation [497].

Since neither P2X6 knockout nor transgenic animals have been generated so far, the in vivo function of the P2X6 receptor remains poorly understood. Nevertheless, in human airway epithelial cells, siRNA-mediated knockdown of P2X6 subunits resulted in significant inhibition of zinc-induced Ca^2+^ entry [465]. As mentioned previously, the same phenotype has been obtained with P2X4-specific siRNA, thus indicating that both subunits may coassemble and function as heteromeric P2X4/P2X6 receptors [465]. Moreover, the re-appearance of P2X6 expression in regenerating muscle fibers from both Duchenne muscular dystrophy patients and dystrophin-deficient mice (mdx) was observed, and an involvement of P2X6 receptors in the regeneration of dystrophic muscles has been suggested [382]. In addition, marked upregulation of the P2X6 subunit in myocardial tissue from chronic heart failure patients was reported, indicating that P2X6 receptors may contribute to the progression of this disease [498]. A role for P2X6 subunits in the differentiation of mesenchymal stem cells has recently been proposed [499].

### P2X7

The cDNA encoding the P2X7 receptor (originally termed P2Z) was first cloned from a rat brain cDNA library and subsequently from different tissues of various species, including human monocytes, mouse microglial cells, *X*. *laevis* stomach, and more recently, guinea pig brain [125, 133, 294, 500, 501]. The P2X7 channel is mainly expressed on cells of hematopoietic origin (monocytes, macrophages, lymphocytes, dendritic cells, mast cells) as well as on different types of glial cells present in the central (microglia, astrocytes, oligodendrocytes, ependymal cells) and the peripheral (Schwann cells, satellite cells, enteric glial cells) nervous systems [125, 332, 369, 449, 502–509]. In addition, it is widely distributed on various epithelial and endothelial cells [381, 510–513]. Although P2X7 mRNA has been detected in neurons and several groups have reported P2X7 antibody staining in neurons [514–520], the presence of P2X7 protein and its function in neurons remains a matter of controversial debate. This is partly due to the poor selectivity of the used antibodies and lack of selective ligands [312, 339, 521]. Experiments on P2X7−/− mouse lines suggest that the antibodies detect an unspecific or “P2X7-like” protein in neurons that could be clearly differentiated from the P2X7 protein by molecular size comparison. The currently identified rodent splice variants or potential rodent orthologues of the identified human splice variants cannot account for these observations.

The gene encoding the P2X7 receptor consists of 13 exons and lies in tandem with the P2X4 gene on human and rat chromosome 12 and murine chromosome 5 [522]. In humans, nine different splice variants, P2X7B-J, have been identified so far [308, 523, 524]. Four of these, P2X7B, P2X7E, P2X7G, and P2X7I, contain a retained intron 10 with a premature stop codon leading to C-terminally truncated P2X7 forms. P2X7G and P2X7H contain an alternative exon N3 with a new start codon that leads to the translation of non-functional P2X7 proteins lacking TM1 [523]. In addition, removal of one or more of the 13 P2X7A exons occurs in some variants [523]. The P2X7J variant is truncated downstream of exon 7 (encoding part of the extracellular loop) and non-functional on its own [524]. In heterooligomeric complexes with P2X7A, it displays dominant negative properties [524] and has been reported to inhibit P2X7A-induced apoptosis, thus contributing to uncontrolled growth of cancer cells. In contrast, hetero-oligomerization of P2X7B with P2X7A has been demonstrated to potentiate P2X7 receptor responses and exert trophic effects [308]. In rodents, three different splice variants have been identified [300, 525]. The fully functional P2X7K variant is derived from an alternative exon 1’, within intron 1 of the rodent P2X7 gene and contains an alternative N terminus and TM1 (Fig. 4). The P2X7K channel shows higher agonist sensitivity, slower deactivation kinetics, and increased pore formation activity [300]. More recently, two novel splice variants in mouse were reported that utilize alternative exons 13b or 13c and encode different C-terminally truncated P2X7 isoforms. The expression pattern of both splice products, but especially P2X713b, has been shown to overlap to a large extent with P2X7A. Moreover, both isoforms exhibited relatively small current responses and poor plasma membrane delivery. The P2X713b isoform was shown to form heteromeric complexes with P2X7A and downregulate its function [525].

Besides alternatively spliced forms, over 650 SNPs have been reported in the human P2X7 gene [526]. Some of these were found to confer loss- or gain-of-function phenotypes, and several of these P2X7 genetic variants have been associated with higher susceptibility to diseases, including infections with intracellular pathogens (e.g., tuberculosis, toxoplasmosis), chronic lymphocytic leukemia, diabetes, and mood disorders [527–538]. However, these genetic associations could not always be replicated across different populations [539–543].

The physiological function of P2X7 receptors has been investigated in three independently generated P2X7−/− mouse models [323, 339, 544, 545]. In the mouse line established by Glaxo, the P2X7 gene was disrupted by targeted insertion of a lacZ/Neo reporter cassette into exon 1, 2 bp downstream of the ATG start codon (Fig. 4) [339, 544, 546]. Later analysis, however, demonstrated that this knockout strategy does not result in complete inactivation of the P2X7 gene since translation of the P2X7K splice variant is not prevented (Fig. 4) [300]. This is in agreement with the observation that T cells from Glaxo P2X7 KO mice exhibit fully functional P2X7 responses [546]. In the mouse line produced by Pfizer, a portion of exon 13, encoding Cys506 to Pro532, has been deleted and replaced with a neomycin resistance cassette (Fig. 4) [323]. Also about this mouse model, concerns have been raised because a C-terminally deleted P2X7 receptor could theoretically still be expressed. Expression of a “P2X7-like” protein has been reported in the brain of these mice and could represent either an unknown P2X7 splice variant or a novel protein, with a similar antibody epitope [547, 548]. In addition, the possibility that the recently described C-terminally truncated variants of the P2X7 receptor (P2X713b, P2X713c) escape the inactivation strategy cannot be excluded either [525].

Despite the described limitations, the existing P2X7 receptor knockout mouse models greatly contributed to our understanding of the physiological function of the P2X7 receptor. The Pfizer P2X7-deficient mice have established an important role for P2X7 receptor-mediated signalling in cytokine production and inflammation [322, 323]. It was demonstrated that LPS-activated macrophages from P2X7−/− mice failed to process pro-IL-1β and consequently did not release mature IL-1β in response to ATP treatment [323]. The same effect was found with ATP-challenged and LPS-primed leukocytes derived from P2X7−/− blood samples [322]. Using a monoclonal antibody-induced arthritis model, it was further demonstrated that P2X7-deficient mice exhibited diminished inflammatory responses and reduced cartilage destruction [322].

In agreement with an important role in inflammation, analysis of the P2X7 KO mice developed by Glaxo [321] showed a complete elimination of hypersensitivity to both inflammatory (intraplantar injection of Freund’s complete adjuvant) and neuropathic (partial ligation of the sciatic nerve) chronic pain states and a marked reduction in mature IL-1β production. A similar effect was also observed for LPS-activated microglia in the dorsal horn of rats [549]. The relevance of P2X7 receptors in the development of neuropathic pain was supported by experiments demonstrating an increase in P2X7 receptor expression in injured nerves from patients suffering from neuropathic pain [321]. Importantly, administration of P2X7-specific antagonists was shown to mimic the knockout phenotype in rodents and thus further established the P2X7 receptor as a therapeutic target [152, 158, 160–162].

In contrast to the above findings, it was recently shown that the P2X7 receptor does not play a role in bone cancer pain. In fact, the Pfizer P2X7 KO mice used in this study exhibited a more severe pain phenotype. This unexpected result can be explained by the different nociceptive mechanism in bone cancer pain that, in contrast to inflammatory or neuropathic pain, does not involve immune cell activation. The results thus underline the important role of microglia activation in neuropathic pain [550, 551].

The Pfizer P2X7−/− mice also developed skeletal abnormalities and revealed the involvement of P2X7 receptors in periosteal bone formation and trabecular bone remodeling [552]. Further studies demonstrated a reduction of sensitivity to mechanical loading in these mice, suggesting a role for P2X7 channels in bone mechanotransduction [553]. Regulation of osteoclast homeostasis by P2X7 receptors has also been demonstrated in vitro. For instance, blocking of the P2X7 receptor with either oxidized ATP or monoclonal antibodies in cultured human osteoclasts resulted in marked inhibition of the mononuclear preosteoclast fusion and their differentiation into multinucleated osteoclasts [554]. In contrast, no bone phenotype has been observed in the Glaxo P2X7 KO animals, suggesting that, in bone tissue, the P2X7K isoform may compensate for the lack of the P2X7A receptor [555].

Analysis of the Pfizer P2X7−/− mice further demonstrated that elimination of P2X7 receptors, in males but not in females, leads to reduced fluid secretion in salivary gland and pancreas but increased secretion in lacrimal glands [556, 557]. Hence, it has been suggested that P2X7 channel might also play a role in the regulation of exocrine gland secretion.

Several studies suggest that P2X7-mediated signalling contributes to neurodegenerative processes observed in CNS diseases, including multiple sclerosis, Alzheimer’s, and Parkinson’s diseases [558–561]. Upregulation of P2X7 receptor expression has frequently been reported in animal models and patients suffering from neurodegenerative conditions [560, 562–564]. An increased expression of P2X7 receptors, altered calcium-signalling, and increased susceptibility to apoptosis was reported in neurons from a mice model of Huntington’s disease [565]. Inhibition of P2X7 receptors in rats was shown to reduce neuronal degeneration and improve recovery after spinal cord injury [566]. Analysis of Glaxo P2X7−/− mice in an induced model of multiple sclerosis demonstrated a markedly reduced incident of disease and highlighted a role for astroglial P2X7 receptors in disease progression [561]. Furthermore, antagonist-mediated P2X7 receptor inhibition resulted in suppression of symptoms associated with the disease [560]. Contrary to these findings, Pfizer P2X7−/− mice were reported to be more susceptible to the disease and exhibited enhanced inflammation in CNS when compared with wild-type controls [567]. Also, in studies on the role of P2X7 receptors in Parkinson’s disease, conflicting results have been obtained [559, 568]. Further studies are needed to validate the existing findings and to confirm the potential of the P2X7 receptor as a therapeutic target for the treatment of neurodegenerative diseases.

Genetic studies showed an association between the Q460R P2X7 polymorphism with mood disorders. [527, 534, 535]. A possible link to P2X7 receptor function is provided by the implication of pro-inflammatory cytokines, including IL-1β in the etiopathogenesis of “sickness behavior” in mice and depression in humans [569–572]. To further test a role of P2X receptors in mood disorders, P2X7−/− mice were assayed in different behavioral models of depression [545, 573]. Using a novel mouse line generated at Lexicon Genetics Inc. (Fig. 4), an anti-depressant-like phenotype was found [545], and in the Pfizer−/− mouse line, an impaired adaptive coping response to repeated stress was observed [573].

It was also reported that P2X7 receptors, by affecting neurotransmitters release, modulate synaptic activity and neuron-glia signalling in the brain. P2X7 receptors on murine cortical astrocytes were shown to contribute to the release of excitatory amino acids, such as glutamate and aspartate [574], and in hippocampal slices from P2X7−/− mice, the ATP-induced efflux of GABA and glutamate was found to be attenuated in comparison to wild-type animals [575]. A more recent study showed induction of IL-1β mRNA expression in the hippocampus after a spatial memory task in wt but not P2X7−/− mice [576]. Another study on P2X7-/- mice suggested the involvement of P2X7 receptors in sleep via release of cytokines and other sleep regulatory substances [577].

P2X7 receptor-mediated signalling has also been postulated to play a role in cancer physiology. Although unusually high levels of P2X7 receptor have been found in diverse tumors and the P2X7 receptor has been proposed as a novel cancer biomarker, its link to cancer remains unclear [488, 509, 578–583]. Nevertheless, some recent studies have demonstrated a positive correlation between P2X7 receptor expression/activation and tumor metastasis [269, 584], and activation of P2X7 receptors was shown to promote invasiveness of aggressive human breast cancer cells [584]. In agreement with this finding, both short hairpin RNA-mediated silencing of P2X7 mRNA or block of P2X7 receptors with antibodies resulted in attenuated metastasis of murine lymphoid neoplasm P388D1 cells [585].

An ortholog of the P2X7 gene has been identified on zebrafish chromosome 8 [429] and two transcript variants, containing 14 and 16 exons, respectively, have been deposited at the Ensembl database. A widespread distribution of the zP2X7 receptor in non-nervous tissues of zebrafish embryo has been reported [429].

Despite such a broad utility of used genetic technologies, the interspecies differences have to be taken into account when translating phenotypic data from animals to humans [80, 526]. Importantly, marked differences in P2X7 receptor function exist between rodents and human and moreover even between different mouse strains [125, 133, 526]. For instance, the naturally occurring allelic polymorphism P451L located in the P2X7 cytoplasmic domain has been shown to significantly impair function of the channel [305]. It has been reported that human, rat, and the BALB/c and 129/Sv mouse strains carry the high-activity variant P451, whereas the C57BL/6 and DBA/2 mouse strains have the low-activity allele L451 [305, 546]. This finding is essential for the interpretation of phenotypic changes observed for example in lymphocytes from P2X7 KO mice [546]. For more detailed information about pharmacological differences between various mammalian P2X7 receptors, see also the section **“** Pharmacological characteristics of P2X receptors” and refer to Donnelly-Roberts et al. [79].

## Concluding remarks

Following the postulation of a class of ATP-gated ion channels about 20 years ago [3], P2X receptors have repeatedly amazed us as a surprisingly unusual class of ligand-gated ion channels. Cloning of the first subtypes and subsequent biochemical and functional analysis of heterologously expressed wt and mutant receptors has revealed a completely novel ion channel structure with various unexpected properties. The long-awaited first crystal structure of a P2X receptor confirmed many of the predictions based on mutagenesis studies and started a new era in which mutations can be planned and results can be explained based on this structure or subtype-specific homology models. Despite this great progress, the movements during channel opening and desensitization are still unclear, and additional crystal structures in the ligand-bound and open states as well as studies investigating the receptor dynamics, such as voltage clamp fluorometry, would greatly help to understand these processes. In particular, the molecular mechanisms underlying subtype-specific molecular functions such as pore dilation and plasma membrane morphology changes remain absolutely enigmatic, and the elucidation of these and other receptor functions that most likely involve interactions with additional and so far mostly unidentified proteins might constitute even more challenging tasks.

Also, the phylogenetic origin of this channel family remains a mystery. Another exciting discovery was that P2X-receptors in evolutionary old organisms serve intracellular functions raising the intriguing and so far hardly addressed possibility of intracellular functions in vertebrates. After many basic principles of P2X receptor function and consequences of their activation have been worked out on the cellular level, the generation of genetically modified animal models now opens opportunities for in vivo studies. For five of the seven P2X receptor subtypes, KO mice have been generated and have confirmed the involvement of these receptors in pathological conditions such as neuropathic pain, inflammation, and thrombosis, to only name a few. P2X transgenic animal models are currently being created. Thus, multiple tools are now emerging that help to decipher the physiological functions of these receptors and their validation as drug targets. Both the advances in understanding the molecular structure and function of these receptors as well as the increasing availability of animal models will greatly accelerate the processes of drug development. However, species differences in the physiology and pharmacology of these receptors as well as the presence of receptor isoforms must be considered and might turn out to be of particular relevance for this puzzling receptor class.
